# MdMYB46 could enhance salt and osmotic stress tolerance in apple by directly activating stress‐responsive signals

**DOI:** 10.1111/pbi.13151

**Published:** 2019-05-26

**Authors:** Keqin Chen, Mengru Song, Yunna Guo, Lifu Liu, Hao Xue, Hongyan Dai, Zhihong Zhang

**Affiliations:** ^1^ Group of Molecular Biology of Fruit Trees College of Horticulture Shenyang Agricultural University Shenyang Liaoning China; ^2^ Group of Fruit Germplasm Evaluation & Utilization College of Horticulture Shenyang Agricultural University Shenyang Liaoning China

**Keywords:** apple, MdMYB46, osmotic stress, salt stress, stress signalling transcription factors

## Abstract

To expand the cultivation area of apple (*Malus×domestica* Borkh.) and select resistant varieties by genetic engineering, it is necessary to clarify the mechanism of salt and osmotic stress tolerance in apple. The MdMYB46 transcription factor was identified, and the stress treatment test of *MdMYB46*‐overexpressing and *MdMYB46*‐RNAi apple lines indicated that MdMYB46 could enhance the salt and osmotic stress tolerance in apple. In transgenic Arabidopsis and apple, MdMYB46 promoted the biosynthesis of secondary cell wall and deposition of lignin by directly binding to the promoter of lignin biosynthesis‐related genes. To explore whether MdMYB46 could coordinate stress signal transduction pathways to cooperate with the formation of secondary walls to enhance the stress tolerance of plants, *MdABRE1A*,* MdDREB2A* and dehydration‐responsive genes *MdRD22* and *MdRD29A* were screened out for their positive correlation with osmotic stress, salt stress and the transcriptional level of *MdMYB46*. The further verification test demonstrated that MdMYB46 could activate their transcription by directly binding to the promoters of these genes. The above results indicate that MdMYB46 could enhance the salt and osmotic stress tolerance in apple not only by activating secondary cell wall biosynthesis pathways, but also by directly activating stress‐responsive signals.

## Introduction

Drought and salt stresses are the two of major environmental factors that affect the geographical distribution of plants in nature, limit plant productivity in agriculture and threaten food security (Zhu, 2001, 2002, 2016). As a consequence of these stresses, plants may suffer photosynthesis inhibition, metabolic dysfunction and cellular structure damage (Krasensky and Jonak, 2012). Apple is a worldwide fruit tree resource and an integral part of a human nutritious diet, but their global cultivation and promotion is limited by drought and high salt stress. So how apple plants sense stress signals and adapt to adverse environments are fundamental biological questions.

Drought mainly led to the osmotic stress while salt stress has both osmotic and ionic or ion‐toxicity effects on cells (Zhu, 2016). The stress resistance of plants is closely related to the formation of secondary cell wall and the deposition of lignin. As the second largest biopolymer in vascular plants (Boerjan *et al*., 2003; Himmel, 2008), the main physiological function of lignin includes providing a structural barrier to the cell wall (Ferrer *et al*., 2008; Sykes *et al*., 2015; Weng and Chapple, 2010), performing basic biological functions such as mechanical support, impermeability and resistant biodegradation, and establishing biological defence mechanism (Bhuiyan *et al*., 2009; Xu *et al*., 2009). The lignin biosynthesis pathway is usually divided into two parts: the common phenylpropane pathway from phenylalanine to feruloyl‐CoA and the specific pathway from feruloyl‐CoA to monolignin units (Vanholme *et al*., 2012), which were regulated by a three‐level regulatory network (Hussey *et al*., 2013; Kumar *et al*., 2015). The regulators of Tier 3 mainly include NST1 (NAC secondary wall thickening promoting factor 1), SND1 (secondary wall thickening promoting factor 1), NST2, VND6 (vascular‐related NAC domain 6) and VND7 transcription factors (TFs), which can regulate the expression of Tier 2 or Tier 1. Tier 2 regulatory factors including MYB family TFs (MYB46, MYB83 and MYB55) and NAC TFs [SND3 and XND1 (xylem NAC domain 1)] (Yamaguchi *et al*., 2011; Zhong *et al*., 2010) can directly regulate the expression of structural genes and Tier 1, which can also directly bind to the cis‐element of structural genes. Recent study found that MdMYB88 and MdMYB124 could regulate root xylem development by directly binding *MdMYB46* promoters and influencing the expression of their target genes under drought condition (Geng *et al*., 2018). In addition to TFs, the metabolic regulatory network of lignin is also regulated by microRNAs (Zhao *et al*., 2015) and Kelch repeat F‐box proteins (Zhang *et al*., 2013). Although the regulatory network of lignin metabolism and its role in plant stress resistance have been paid more attention, the related research is mostly carried out in herbaceous plants such as *Arabidopsis thaliana*, and the related research on fruit trees, such as apple, is still lacking.

MYB46, as the direct target of SND1, plays a key regulator in the transcriptional network involved in the regulation of secondary wall biosynthesis in *Arabidopsis* (Zhong *et al*., 2007). Although its role in regulating secondary wall formation and its regulation mode of activating gene expression by binding elements such as SMRE, MYBCORE and AC‐box in the promoter region have been elucidated (Nakano *et al*., 2015; Zhong and Ye, 2011), its function in other metabolic (such as disease susceptibility) and signal transduction pathways in plants is still being explored (Ramírez *et al*., 2011). In recent years, *MYB46* was found to be activated under stress (Geng *et al*., 2018; Shafi *et al*., 2019), and it was put forward that BplMYB46 might have the function of regulating the expression of genes involved in abiotic stress responses of *Betula platyphylla* (Guo *et al*., 2016). However, the genes and metabolic pathways that could be specifically regulated by MYB46 under stress remain to be further explored and determined.

Previous studies showed that several MYB proteins, such as AtMYB2, AtMYB14, AtMYB15 and AtMYBS3, could affect the stress tolerance of plants by regulating the expression of stress‐responsive genes (Abe *et al*., 2003; Agarwal *et al*., 2006a; Chen *et al*., 2013; Su *et al*., 2010). Osmotic stress can induce a series of signal transduction and response reactions in plants (Zhu, 2002, 2016), some of which are controlled by abscisic acid (ABA) but some are not, in other words, both ABA‐dependent and ABA‐independent regulatory systems are involved in stress‐responsive process (Yamaguchi‐Shinozaki and Shinozaki, 2005). The cis‐acting element, ABA‐responsive element (ABRE) and a group of TFs, ABRE‐binding protein/ABRE‐binding factors (AREB/ABFs) have pivotal functions in regulating ABA‐dependent gene expression, while the cis‐element, dehydration‐responsive element/C‐repeat (DRE/CRT) and DRE‐/CRT‐binding protein 2 (DREB2) TFs play key roles in regulating ABA‐independent gene expression in response to osmotic stress (Abe *et al*., 2003; Agarwal *et al*., 2006a,b; Lata and Prasad, 2011; Thomashow, 2010; Yoshida *et al*., 2014). Using MYB46 as the entry point, we carried out research on the regulation network of secondary cell wall formation and the signal transduction mechanism to salt and osmotic stress in apple (*Malus×domestica* Borkh.). And we found that MdMYB46 can activate not only the expression of genes involved in the biosynthesis of secondary cell wall, but also ABA‐dependent and independent stress signals. In the aspect of improving plant stress tolerance, the biosynthesis of secondary cell wall and stress signal transduction pathways of plants were coordinated by MdMYB46.

## Results

### MdMYB46 protein has the R2 and R3 functional regions

The MYB transcription factor gene *MdMYB46* [numbered XP_008363629 in National Center for Biotechnology Information or MD03G1176000 in the Apple Genome (GDDH13 V1.1) database] was isolated from the wild GL‐3 apple (*M. ×domestica* Borkh). Multiple sequence alignments and phylogenetic analysis indicated that MdMYB46 was an R2R3‐type protein and in the same subfamily with MtMYB46, GmMYB46A, GmMYB46B and VvMYB46A among those AtMYB46 orthologs (Figure S1).

### MdMYB46 with transcriptional activation activity can be activated by stress signals

**Figure 1:**
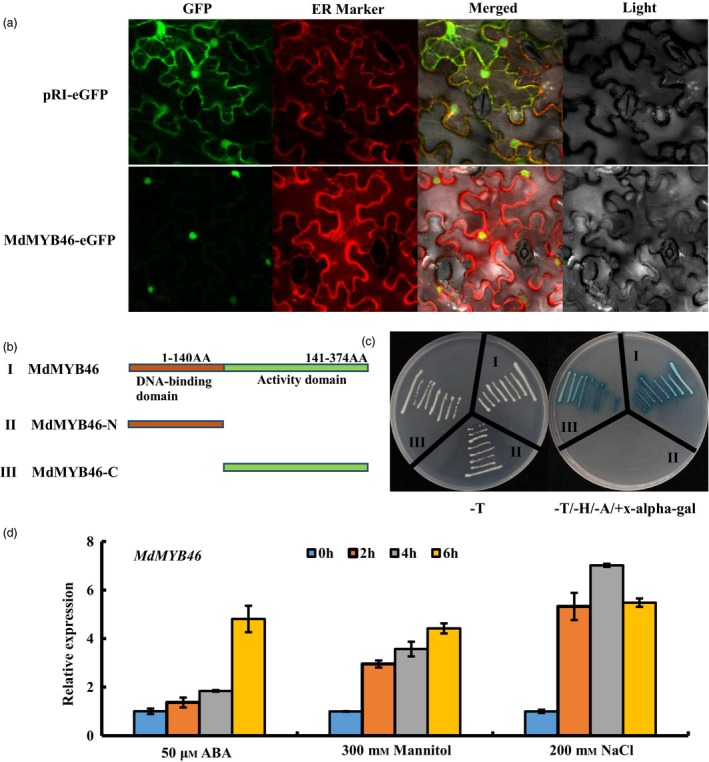
Subcellular localization and transcriptional activation of the MdMYB46 from apple. (a) Colocalization of pRI‐MdMYB46‐eGFP in tobacco epidermal cells. (b) The structure of full‐length MdMYB46, MdMYB46‐N terminal and MdMYB46‐C terminal. (c) Transcriptional activation assays of full‐length MdMYB46, MdMYB46‐N terminal and MdMYB46‐C terminal fused with the GAL4 DNA‐binding domain (GAL4DB) in yeast. (‐T) indicates selective medium lacking Trp, (‐T/‐H/‐A+X‐alpha‐gal) indicates selective medium lacking Trp, His, adenine and plus 5‐Bromo‐4‐chloro‐3‐indolyl‐α‐D‐galactoside. (d) *MdMYB46* expression levels in non‐transformed apple plants treated with ABA, NaCl or mannitol. [Colour figure can be viewed at wileyonlinelibrary.com]

To elucidate the localization pattern of MdMYB46 in cells, the CDS region (without stop codon) was inserted into the pRI‐eGFP vector to construct a MdMYB46‐eGFP fusion gene, which was then transiently expressed by infiltration in tobacco leaves. As shown in Figure 1a, the localization results showed that MdMYB46 localized to the nucleus. Using the yeast two‐hybrid system, the transcriptional activity of MdMYB46 was validated and the specific activity region was analysed. It was found that MdMYB46 has transcriptional activation activity at the C‐terminal position of 141–374 amino acids (Figure 1b,c).

In order to clarify the expression patterns of *MdMYB46* under abiotic stress, non‐transgenic GL‐3 apple plants were treated with exogenous ABA, NaCl and mannitol. QRT‐PCR results showed that the expression level of *MdMYB46* was up‐regulated under these stresses (Figure 1d).

### MdMYB46 positively regulates salt and osmotic stress tolerance in apple

In order to verify the function of MdMYB46 in apple, we constructed the overexpression and RNAi vectors of *MdMYB46* with the CaMV 35S fragment in the promoter (Figure S2A). Four *MdMYB46*‐overexpressing transgenic lines (OE‐MdMYB46‐3, OE‐MdMYB46‐9, OE‐MdMYB46‐14 and OE‐MdMYB46‐25) and four MdMYB46‐RNAi lines (RNAi‐MdMYB46‐7, RNAi‐MdMYB46‐8, RNAi‐MdMYB46‐15 and RNAi‐MdMYB46‐19) were obtained with *Agrobacterium*‐mediated transformation. Compared with the non‐transgenic apple plants, the expression levels of *MdMYB46* were significantly increased in the four *MdMYB46*‐overexpressing lines (Figure S2B), while those in four *MdMYB46*‐RNAi lines were significantly decreased (Figure S2B).

**Figure 2:**
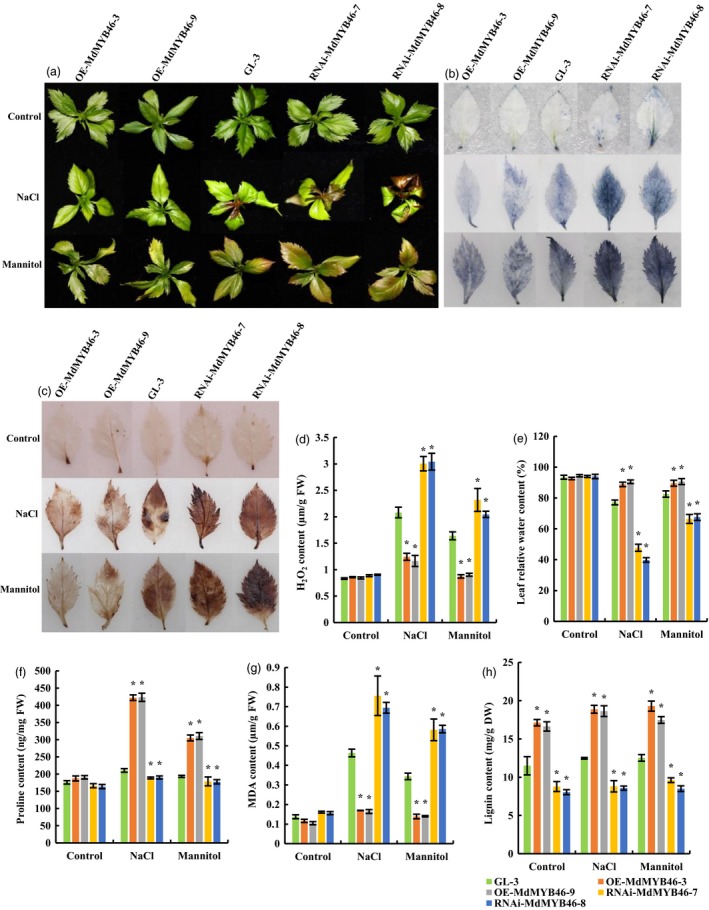
Stress tolerance of transgenic apple plants under salt and osmotic stress. (a) Phenotypic comparison of *MdMYB46*‐overexpressing and *MdMYB46*‐RNAi apple plants subjected to salt and osmotic stress. Twenty‐day‐old non‐transgenic GL‐3 apple plants (WT) and transgenic apple plants were grown under 200 mm NaCl or 300 mm mannitol stress for 10 days. (b) Nitroblue tetrazolium (NBT) staining of apple leaves from plants subjected to salt and mannitol treatments for 10 days. (c) 3, 30‐diaminobenzidine (DAB) staining of apple leaves from plants subjected to salt and mannitol treatments for 10 days. (d) Content of H_2_O_2_. (e) Relative water content. (f) Proline content. (g) Malondialdehyde (MDA) content. (h) Lignin content. The error bars indicate the standard deviation (SD) from three biological replicates. Asterisk indicates significant differences between the transgenic lines and GL‐3 (*P *<* *0.05, based on *t*‐test). [Colour figure can be viewed at wileyonlinelibrary.com]

The transgenic apple lines of OE‐MdMYB46‐3, OE‐MdMYB46‐9, RNAi‐MdMYB46‐7, RNAi‐MdMYB46‐8 and the non‐transgenic apple plants (GL‐3) were chosen for long‐time stress treatment. It was found that *MdMYB46*‐overexpressing lines were more resistant to salt and osmotic stress than the non‐transgenic plants, while *MdMYB46*‐RNAi lines were less resistant (Figure 2a). The phenotypic differences between each group were especially obvious under the high salt treatment. Compared with the leaves of non‐transgenic apple, the leaves of *MdMYB46*‐overexpressing plants, with the normal leaf morphology, were greener, while the leaves of *MdMYB46*‐RNAi plants were yellowish brown and severely curled under salt treatment. Similarly, the leaves of *MdMYB46*‐overexpressing plants were still green or slightly yellow after mannitol stress treatment, while the leaves of *MdMYB46*‐RNAi plants became brown.

Plants produce reactive oxygen species under stress conditions. H_2_O_2_ and superoxide (O_2_^−^) anions (both of which are most important reactive oxygen species) in leaves can be histochemical stained with DAB and NBT. As a chromogenic substrate, DAB can be oxidized by H_2_O_2_ in the presence of peroxidase to produce a reddish brown precipitate, and NBT can react with O_2_^−^ to form a deep blue insoluble compound. The staining results of DAB and NBT in this study indicated that the content of active oxygen in *MdMYB46*‐overexpressing apple plants under salt and osmotic stress was lower than that in non‐transgenic plants, while the level of active oxygen in leaves of *MdMYB46*‐RNAi plants was higher (Figure 2b,c). Meanwhile, the quantitative results showed that the content of H_2_O_2_ in leaves of *MdMYB46*‐overexpressing plants was lower than that in the non‐transgenic plants under salt and osmotic stress, while that in *MdMYB46*‐RNAi plants was the highest (Figure 2d).

The relative water content of the leaves can be used as an indicator of plant water loss. This study also examined the relative water content in leaves of transgenic and non‐transgenic apple plants under salt and osmotic stress (Figure 2e). The results showed that under salt and osmotic stress, the water loss in *MdMYB46*‐overexpressing apple leaves was the least, while that in the RNAi plants was the highest. Proline content could reflect the stress resistance of plants to a certain extent. With the highly hydrophilic, proline can stabilize the metabolic processes in cells and tissues, thus reducing the freezing point and effectively preventing cell dehydration. Plants with strong osmotic stress resistance often accumulate more proline. In this study, under the stress of salt and osmotic, the content of proline in the leaves of *MdMYB46‐*overexpressing transgenic apple plants was higher than that in non‐transgenic plants, while that in the RNAi plants was the lowest (Figure 2f).

Under adverse conditions, plant membrane lipid peroxidation often occurs. MDA is one of its products. It is usually used as an indicator of lipid peroxidation, indicating the degree of cell membrane lipid peroxidation and the strength of plant response to stress conditions. In this study, under the stress of salt and osmotic, the content of MDA in the leaves of *MdMYB46‐*overexpressing transgenic apple plants was lower than that of non‐transgenic plants, while that in the RNAi plants was the highest (Figure 2g).

The content of lignin in the transgenic plants showed significant changes (Figure 2h). The content of lignin in the *MdMYB46*‐overexpressing plants was significantly higher than that in non‐transgenic and *MdMYB46*‐RNAi apple plants, indicated that MdMYB46 could significantly regulate the accumulation of lignin. Under osmotic stress, the lignin content in apple plants increased, but the increasing amount was less in non‐transgenic and *MdMYB46*‐RNAi plants than that in the overexpressing plants.

The above experimental results indicated that *MdMYB46*‐overexpressing apple plants had higher salt and osmotic stress tolerance than the non‐transgenic plants, while *MdMYB46*‐RNAi transgenic apples had weaker salt and osmotic stress resistance.

### The transcript levels of secondary cell wall biosynthesis‐related genes in apple and arabidopsis are induced by MdMYB46

The results of stress test showed that *MdMYB46*‐overexpressing apple plants were more resistant to osmotic stress. Previous studies have shown that MYB46 could enhance the resistance of plants to abiotic stress by regulating the formation of secondary walls of plant cells (Guo *et al*., 2016; Zhong *et al*., 2007). Therefore, we explored the function of MdMYB46 in the regulation of secondary cell wall, in which the transgenic Arabidopsis and transgenic apples were used.

With overexpressing *MdMYB46* (Figure S4), Arabidopsis plants showed obviously change in phenotype. Compared with the wild type, *MdMYB46*‐overexpressing Arabidopsis plants not only showed short stature, but also showed the characteristics of blade curling (Figure S3A) and floral organ development abnormalities (Figure S3B), which may be caused by the ectopic lignin deposition into epidermal or mesophyll cells (Zhong *et al*., 2007). However, it was so different in *MdMYB46*‐overexpressing apple plants, of which the size was similar to the non‐transgenic plants (Figure S3C).

**Figure 3:**
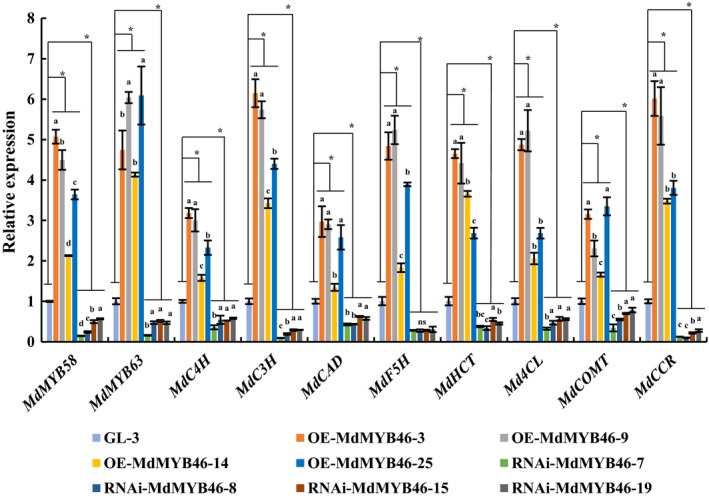
Transcriptional levels of lignin biosynthesis‐related genes in *MdMYB46* transgenic apples. The error bars indicate the standard deviation (SD) from three biological replicates. Asterisk indicates significant differences between GL‐3 and the OE‐MdMYB46 lines or RNAi‐MdMYB46 lines (*P *<* *0.05, based on *t*‐test). Different letters indicate the different significance in gene expression among the different lines (*P *<* *0.05, based on Duncan's multiple range test). [Colour figure can be viewed at wileyonlinelibrary.com]

To further investigate the regulation network of MdMYB46, the transcriptional levels of MYB transcription factors MYB58, MYB63 and structural genes related to lignin and cellulose biosynthesis in transgenic plants were examined. The expression levels of *MdMYB58*,* MdMYB63*, lignin biosynthesis genes (*MdC4H*,* MdC3H*,* MdCAD*,* MdF5H*,* MdHCT*,* Md4CL*,* MdCOMT* and *MdCCR*) and cellulose biosynthesis genes (*MdCESA4* and *MdCESA8*) were significantly up‐regulated and down‐regulated in the *MdMYB46*‐overexpressing and ‐RNAi lines, respectively (Figures 3 and S5A). In *MdMYB46*‐overexpressing lines of Arabidopsis, the expression levels of *AtMYB63*,* AtMYB58*,* At4CL1*,* AtCCoAOMT*,* AtCESA4*,* AtCESA7* and *AtCESA8* were significantly higher than those in the wild type (Figures S4 and S5B–D).

The above results indicated that MdMYB46 positively regulated the transcriptional levels of secondary cell wall biosynthesis‐related genes and lignin deposition in apple and Arabidopsis.

### MdMYB46 binds to M46RE and SMRE motifs in promoters of lignin biosynthesis‐related genes

Lignin is the phenolic component of woody cell walls and plays the main role in providing compression strength to cell walls. In order to clarify the pattern of MdMYB46 in promoting the deposition of lignin in the secondary cell walls, we analysed the promoter region of lignin biosynthesis‐related genes and specially validated the binding sites for genes involved in osmotic stress.

**Figure 4:**
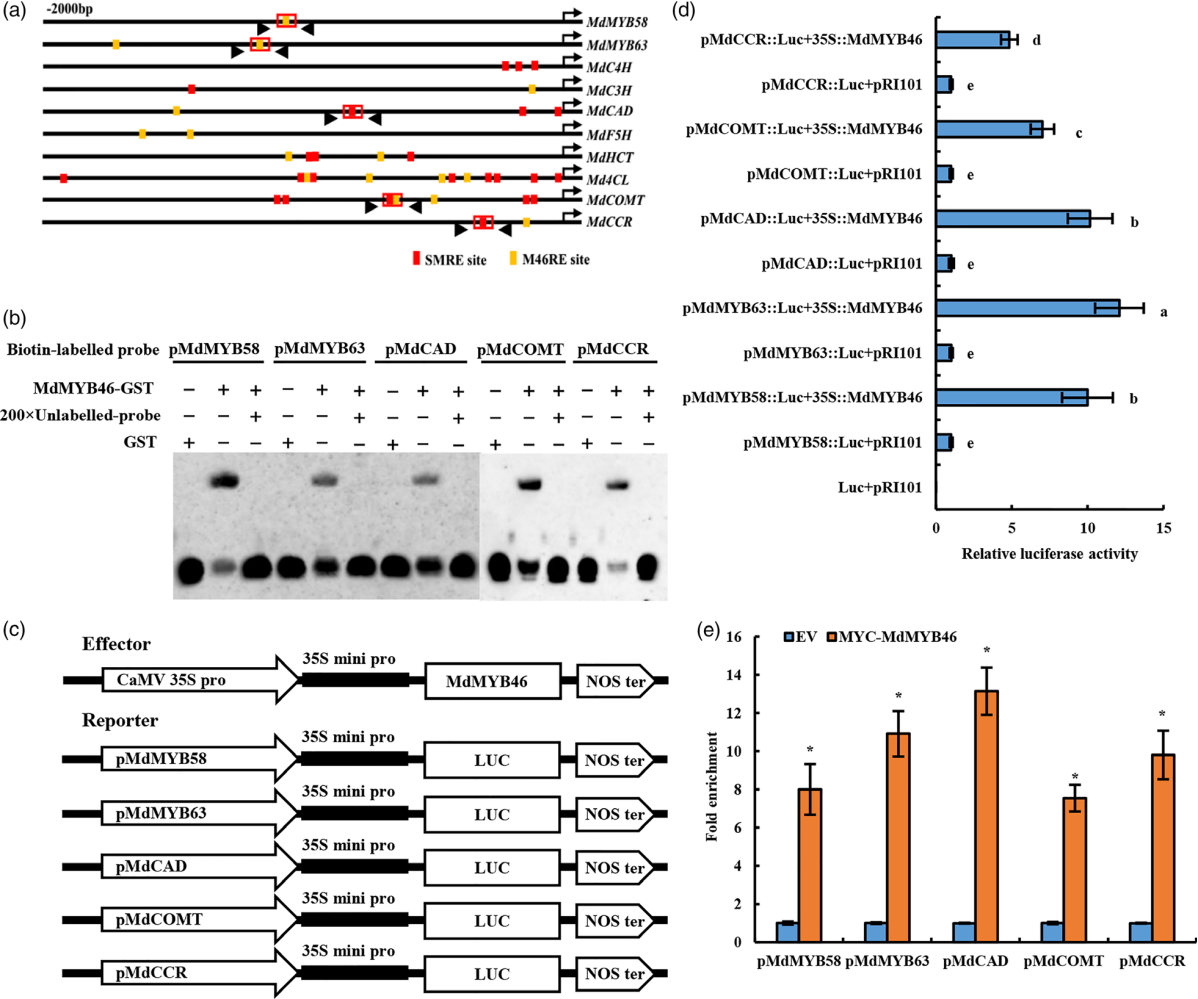
The binding of MdMYB46 to the M46RE and SMRE motifs in the promoter of lignin biosynthesis‐related genes. (a) Schematic representation of lignin biosynthesis gene promoters showing the position of MdMYB46 binding sites. (b) EMSAs performed using probes specific to lignin biosynthesis genes. (c) Schematic diagrams of the effector and reporter constructs used for tobacco transient expression assay. (d) The detection of luciferase activity. Different letters indicate significant differences (*P *<* *0.05, based on Duncan's multiple range test). (e) ChIP‐qPCR assay of MdMYB46 binding to the promoter of the lignin‐related genes. Asterisk indicates significant differences (*P *<* *0.05, based on *t*‐test). The error bars indicate the standard deviation (SD) from three biological replicates. [Colour figure can be viewed at wileyonlinelibrary.com]

MYB46 could specifically bind to the SMRE and M46RE site in its downstream gene promoter. Sequence analysis of promoters of lignin regulatory genes (*MdMYB58*,* MdMYB63*) and biosynthetic genes (*MdC4H*,* MdC3H*,* MdCAD*,* MdF5H*,* MdHCT*,* Md4CL*,* MdCOMT* and *MdCCR*) revealed that multiple MYB46 binding sites exited there (Figure 4a).

MYB58 and MYB63 are transcriptional activators that specifically promote lignin biosynthesis during Arabidopsis secondary wall formation and are also direct target genes for MYB46 (Demura and Ye, 2010; Wang and Dixon, 2012; Zhou *et al*., 2009). *CAD*,* COMT* and *CCR* are the key genes in the biosynthesis pathway of lignin monomers, which are significantly positively correlated with the drought/osmotic stress resistance of plants (Fan *et al*., 2006; Hu *et al*., 2009). So, the study mainly focused on the in‐depth study of the regulation of MYB46 on the expression of these genes.

In order to detect the specificity of MYB46 binding to lignin regulatory genes and biosynthetic gene promoters, biotinylated probes of M46RE and SMRE elements were designed according to the elements in the red box of Figure 4a, and incubated with GST‐MdMYB46 fusion protein. The interaction between the labelled probes and the GST‐MdMYB46 fusion protein was tested by EMSA. The results showed that GST‐MdMYB46 was able to interact with the active elements in the promoters of *MdMYB58*,* MdMYB63*,* MdCAD*,* MdCOMT* and *MdCCR* genes (Figure 4b).

Meanwhile, the fragments containing the SMRE site and the M46RE site (about 200–500 bp) in the promoters of *MdMYB58*,* MdMYB63*,* MdCAD*,* MdCOMT* and *MdCCR g*enes were inserted into the vector containing the luciferase reporter gene (these fragments shown by the black arrow in Figure 4c). p35S:MdMYB46 was used as an effector to inject with pRI‐LUC, pMdMYB58‐LUC, pMdMYB63‐LUC, pMdCAD‐LUC, pMdCOMT‐LUC and pMdCCR‐LUC, respectively. After 48 h, fluorescence was observed and the luciferase activity was detected in tobacco leaves. These results indicated that MdMYB46 could activate the transcription of the lignin‐related genes (Figure 4d).

To further determine the effect of MdMYB46 on lignin‐related genes, ChIP‐qPCR primers were also designed in this experiment (shown by the black arrow in Figure 4a). The binding ability of MdMYB46 to the promoter of lignin biosynthesis genes was detected *in vivo* by transgenic pRI‐MYC‐MdMYB46 ‘Ourin’ apple callus. The result of ChIP‐qPCR is shown in Figure 4e and indicated that MYC‐MdMYB46 could bind to the promoters of *MdMYB58*,* MdMYB63*,* MdCAD*,* MdCOMT* and *MdCCR* genes and directly participate in the regulation of lignin synthesis.

### The expression of some stress‐responsive genes involved in ABA‐dependent or ABA‐independent pathways is induced by MdMYB46 in apple

In order to clarify the role of MdMYB46 in the regulation network of stress tolerance in apple, non‐transgenic apple plants were treated with exogenous ABA, NaCl and mannitol, and the previously reported drought and salt stress related genes *AREB1* (Yoshida *et al*., 2014, 2015), *RD22* (Abe *et al*., 2003) in ABA‐dependent pathway and *DREB* (Agarwal *et al*., 2006b; Lata and Prasad, 2011; Thomashow, 2010), *RD29* (Yamaguchi‐Shinozaki and Shinozaki, 1994) in the ABA‐independent pathway were chosen for our study. The amino sequences in the Apple Genome (GDDH13 V1.1) protein database with highest similarity to AtAREB1A, AtAREB1B, AtDREB2A, AtDREB2B, AtRD29A, AtRD29B and AtRD22 in Arabidopsis were identified and named MdAREB1A, MdAREB1B, MdDREB2A, MdDREB2B, MdRD29A, MdRD29B and MdRD22 accordingly. In apple, some studies have also explored the involvement of these genes in stress signal transduction (Table S4). From Figure S6, it could be found that the expression level of these genes was significantly increased under the stress of exogenous ABA, NaCl and mannitol, except for *MdDREB2B*.

**Figure 5:**
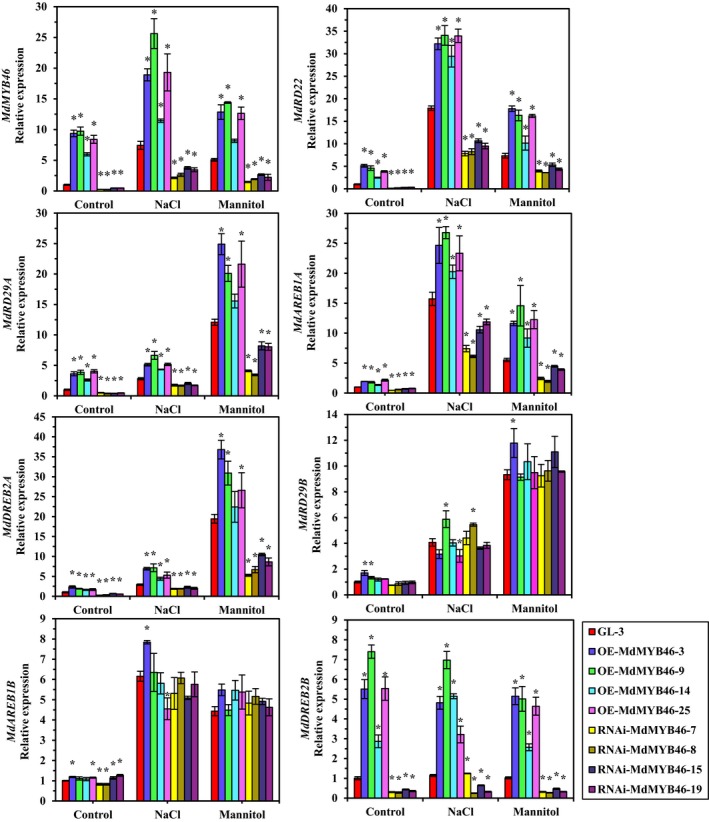
Transcriptional levels of stress signalling genes under short‐time salt and osmotic stress in *MdMYB46* transgenic apples. NaCl means 200 mm NaCl stress, mannitol means 300 mm mannitol stress. Young apple tissues were taken for RNA extraction after 4‐h treatment in NaCl stress or after 6‐h treatment in mannitol stress. The error bars indicate the standard deviation (SD) from three biological replicates. Asterisk indicates significant differences between the transgenic lines and GL‐3 (*P *<* *0.05, based on t‐test). [Colour figure can be viewed at wileyonlinelibrary.com]

To investigate the effects of MdMYB46 on the expression of *MdRD22*,* MdRD29A*,* MdRD29B*,* MdAREB1A*,* MdAREB1B*,* MdDREB2A* and *MdDREB2B*, the transcriptional levels of these genes were studied in the *MdMYB46*‐overexpressing and *MdMYB46*‐RNAi plants without stress treatment (the control in Figure 5). The results showed that the expression level of *MdRD22*,* MdRD29A*,* MdAREB1A*,* MdDREB2A* and *MdDREB2B* genes was positively correlated with the expression level of *MYB46* in apple plants, while the expression levels of *MdRD29B* and *MdAREB1B* changed insignificantly in the overexpressing and RNAi plants, respectively.

The transcriptional levels of stress‐responsive genes in transgenic apple plants under salt and osmotic stress were also studied. After short‐time stress treatment, the transcription levels of *MdRD22*,* MdRD29A*,* MdAREB1A* and *MdDREB2A* showed similar trends with *MdMYB46* (Figure 5). Compared with the control, the expression of the four genes in *MdMYB46*‐overexpressing and *MdMYB46*‐RNAi plants increased after stress treatment, but the expression levels of these genes in RNAi plants were lower than those in the non‐transgenic and *MdMYB46*‐overexpressing plants, indicating that MdMYB46 can regulate the expression of these genes. After stress treatment, the expression levels of *MdRD29B* and *MdAREB1B* increased, but there was no significant difference in the expression level of these genes in *MdMYB46*‐overexpressing and *MdMYB46*‐RNAi plants, indicating that there are still other regulatory factors in apple involved in the ABA‐dependent and ABA‐independent pathway for stress responding.

### MdMYB46 activates the expression of stress signalling genes by binding to SMRE,M46RE and MYBCORE motifs present in their promoters

**Figure 6:**
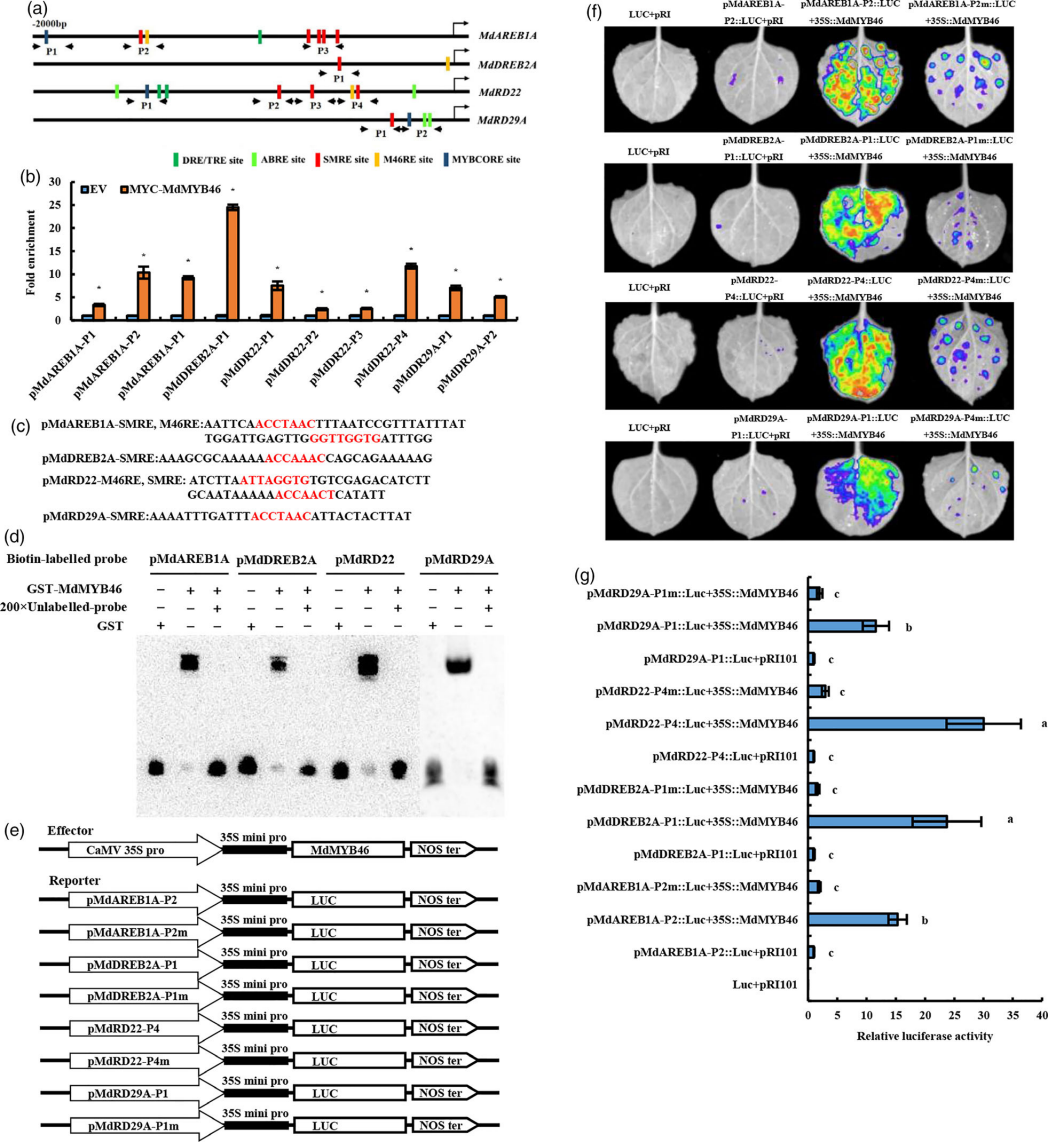
Identification of SMRE,M46RE and MYBCORE motifs in the promoter of stress signalling genes and the activation effect of MdMYB46 on their transcription. (a) Schematic representation of *MdRD22*,* MdRD29A*,* MdAREB1A* and *MdDREB2A* gene promoters showing the position of MdMYB46 binding sites. (b) ChIP‐qPCR assay of MdMYB46 binding to the promoter of *MdRD22*,* MdRD29A*,* MdAREB1A* and *MdDREB2A* genes. Asterisk indicates significant difference (*P *<* *0.05, based on t‐test). (c) The SMRE and M46RE sequence in the MdAREB1A‐P2, MdAREB2A‐P1, MdRD22‐P4 and MdRD29A‐P1 fragments with the strongest binding ability. (d) EMSAs performed using probes specific to *MdRD22*,* MdRD29A*,* MdAREB1A* and *MdDREB2A* genes. (e) Schematic diagrams of the effector and reporter constructs used for tobacco transient expression assay. (f) Luciferase activity assay. (g) The comparison of luciferase activity. Different letters indicate significant differences (*P *<* *0.05, based on Duncan's multiple range test). The error bars indicate the standard deviation (SD) from three biological replicates. [Colour figure can be viewed at wileyonlinelibrary.com]

With the exclusion of genes that were not significantly associated with stress induction or *MdMYB46* expression, the promoter sequences (2000 bp) of *MdRD22*,* MdRD29A*,* MdAREB1A* and *MdDREB2A* were analysed. As shown in Figure 6a, there were five SMRE sites, one M46RE site and one MYBCORE site in the promoter of *MdAREB1A*; one SMRE site and one M46RE site in the promoter of *MdDREB2A*; three SMRE sites, one M46RE site and one MYBCORE site in the promoter of *MdRD22*; and one SMRE site and one M46RE site in the promoter of *MdRD29A*, which indicated that they all had the potential to be activated by MdMYB46. In addition, different from the promoter sequences of *AtRD22* and *AtRD29A* (Yamaguchi‐Shinozaki and Shinozaki, 1993, 1994), two DRE sites were found in the promoter of *MdRD22*, but not in *MdRD29A*, and two ABRE sites were found both in the promoter of *MdRD22* and *MdRD29A* which indicated that a different regulatory pathway of these genes may be existed in apple.

To explore the interaction of MdMYB46 with the MYB binding site in the promoters of *MdRD22*,* MdRD29A*,* MdAREB1A* and *MdDREB2A*, ChIP‐qPCR primers were designed in the black arrow of Figure 6a. The binding signal of SMRE, M46RE and MYBCORE sequence in each gene promoter was detected in transgenic callus containing pRI101‐MYC empty vector or pRI‐MYC‐MdMYB46 vector and showed that MdMYB46 bound to these sites with varying degrees (Figure 6b).

Based on the results of ChIP‐qPCR, the SMRE and M46RE sequences (Figure 6c) in the *MdAREB1A‐P2*,* MdAREB2A‐P1*,* MdRD22‐P4* and *MdRD29A‐P1* fragments with the strongest binding ability were selected to design biotin labelling probes. These probes were then incubated with the GST‐MdMYB46 protein. A 200‐fold unlabelled probe was used as a competition assay, and a protein gel migration assay was used to detect the binding between MdMYB46 and these promoters. Figure 6d indicates that the MdMYB46 protein could be directly bound to the promoters of *MdRD22*,* MdRD29A*,* MdAREB1A* and *MdDREB2A* genes.

To further demonstrate the interaction between MdMYB46 and the promoters of these genes, the fragments of *MdAREB1A‐P2*,* MdAREB2A‐P1*,* MdRD22‐P4*,* MdRD29A‐P1* and the corresponding mutation‐binding site sequences were inserted into the luciferase‐containing reporter gene vector (Figure 6e), which was then injected into the tobacco leaves together with the effector p35S:MdMYB46 and pRI101AN empty vectors, respectively. The fluorescence distribution and the luciferase activity were detected 48 h later (Figure 6f–g). The results showed that MdMYB46 could directly bind to the promoters of *MdRD22*,* MdRD29A*,* MdAREB1A* and *MdDREB2A*.

## Discussion

### MdMYB46 regulates the biosynthesis of secondary cell wall and deposition of lignin in apple

With the evolution of plants and lignin biosynthesis pathways, complex and sophisticated lignin synthesis regulatory networks have been formed in plants. NST1, SND1, NST2, VND6 and VND7 are the main upstream regulators of lignin biosynthesis pathway, while MYB transcription factors (MYB46, MYB83 and MYB55) and NAC transcription factors (SND3 and XND1) (Yamaguchi *et al*., 2011; Zhong *et al*., 2010) belong to the Tier 2 regulators in the regulatory network (Kumar *et al*., 2015). In Arabidopsis, the transcription factor MYB46 showed to be a key transcriptional switch controlling Arabidopsis secondary cell wall biosynthesis (Ko *et al*., 2009; Zhong *et al*., 2007), which can act directly on the M46RE cis‐regulatory element ([A/G][G/T]T[A/T]GGT[A/G]) or SMRE element (secondary wall MYB‐responsive element, ACC[A/T]A[A/C][T/C]), to activate the expression of related genes and regulate lignin synthesis (Kim *et al*., 2012; Ko *et al*., 2009; Zhong and Ye, 2015). In this study, multiple MYB46 binding sites were present in the promoters of the regulatory (*MdMYB58* and *MdMYB63*) and biosynthetic (*MdC4H*,* MdC3H*,* MdCAD*,* MdF5H*,* MdHCT*,* Md4CL*,* MdCOMT* and *MdCCR*) genes for apple lignin metabolism (Figure 3), which was consistent with previous findings in Arabidopsis (Kim *et al*., 2012, 2014; Zhong *et al*., 2007). The EMSA, luciferase reporter assays and ChIP further indicated that MdMYB46 could directly bind to the promoters of *MdMYB58*,* MdMYB63*,* MdCAD*,* MdCOMT* and *MdCCR* to activate the expression of these genes.

In our transgenic Arabidopsis, the overexpression of *MdMYB46* caused a dwarf plant phenotype (Figure S3A) similar to the previous findings in *AtMYB46*‐overexpressing Arabidopsis (Zhong *et al*., 2007), which indicated that MdMYB46 and AtMYB46 had certain functional conservation. But in apple, there was no obvious difference in morphology between the non‐transgenic and transgenic plants, similar to that in the *BplMYB46*‐overexpressing birch (Guo *et al*., 2016). The inconsistency of phenotype between *MYB46*‐overexpressing Arabidopsis and apple plants may be related to the differences between herb and woody plants. Apple trees and birch trees belong to woody plants with higher body size, and their lignin metabolism regulation network may be more complicated and less likely to be destroyed by exogenously introduced genes. Although there was no significant difference in phenotype, the content of lignin increased in *MdMYB46*‐overexpressing apple plants. Due to the main role in providing compression strength to cell walls (Kumar *et al*., 2015), the extra deposition of lignin and formation of secondary cell wall may increase the cell stability under osmotic stress.

### MdMYB46 enhances the salt and osmotic stress tolerance of apple plants by directly activating ABA‐dependent and ABA‐independent stress signals

Extreme drought and high salt stress usually produce high osmotic stress causing dehydration and even death of plant cells, which significantly affect plant growth and development, leading to severe crop yield loss (Agarwal *et al*., 2012; Chaves *et al*., 2009). The salt and osmotic stress tolerance of plants are the results of interaction between genotype and environment. Lignin deposition is related to osmotic stress and salt tolerance of plants and many studies have shown that lignin biosynthesis is enhanced under osmotic stress. Lignin can reduce plant cell wall water leaching and transpiration, which helps maintain cell osmotic balance and protects membrane integrity (Monties and Fukushima, 2005). In addition to the accumulation of lignin, salt and osmotic stress also trigger a series of stress‐responsive pathways in plants. AREB/ABFs are a family of ABA‐dependent bZIP TFs that interact with the ABRE (PyACGTGG/TC) motif in promoter of many ABA‐responsive genes in many plant species (Kang *et al*., 2002; Yoshida *et al*., 2010, 2014). Among the nine *AREB/ABF* genes identified in Arabidopsis, *AREB1/ABF2*,* AREB2/ABF4* and *ABF3* can be induced by osmotic stress. DREB proteins, belonging to AP2/ERF TF family, are indeed implicated in plant responses to drought/osmotic stress tolerance (Lata and Prasad, 2011; Thomashow, 2010), of which DREB1A and DREB2A specifically interact with cis‐acting dehydration‐responsive element (C‐repeat) involved in drought stress‐responsive gene expression in *A. thaliana* (Sakuma *et al*., 2006). Several stress‐responsive genes, such as *RD29A* induced through ABA‐independent pathway and *RD22* mediated by ABA, can be activated by dehydration, high salinity and cold stress (Yamaguchi‐Shinozaki and Shinozaki, 1993, 1994). In apple, some studies have explored the involvement of these genes in stress signal transduction (Table S4). Sixty‐eight *MdDREB* genes have been classified into six subgroups against the entire genome of apple, of which *MdDREB2A* and *MdDREB2B* (called MDP0000147009 and MDP0000153866 in their study) were identified and found they could be up‐regulated under various abiotic stress treatments (Zhao *et al*., 2012). *MdAREB1A* and *MdAREB1B* (called *MdAREB3.2* and *MdAREB2* in their study) were identified and studied for their sensitivity to ABA in apple (Ma *et al*., 2017). *MdRD22*,* MdRD29A* and *MdRD29B* were also found to be responsive for drought or osmotic stress (An *et al*., 2018; Shao *et al*., 2019). These results indicated that these genes in apple were also active in stress signal transduction.

The *MdMYB46*‐overexpressing plants had higher salt and osmotic stress tolerance with lower levels of reactive oxygen species and membrane lipid peroxidation products such as MDA, and higher relative water content and proline content in leaves compared with the non‐transgenic apple plants, while the *MdMYB46*‐RNAi plants had lower resistance with higher level of active oxygen, lipid peroxidation and water loss. These results indicated that in addition to promoting lignin deposition, MdMYB46 might also increase the strength of other stress‐responsive pathways to enhance the stress tolerance of apple plants.

MYB proteins are involved in ABA and stress responses as well as many other cellular processes (Chen *et al*., 2006). In Arabidopsis, *AtMYB52* was regulated by AtMYB46 directly during secondary wall synthesis, and overexpression of *AtMYB52* could improve plant drought tolerance by regulating ABA signalling pathway in which *AtRD29B* and *AtNCED3* may be involved (Ko *et al*., 2009; Park *et al*., 2011). AtMYB2 protein also functioned as a transcriptional activator in ABA‐regulated gene expression under drought and salt stress, and ABA‐induced gene expression of *AtRD22* and *AtADH1* was enhanced in AtMYB2 transgenic plants (Abe *et al*., 2003). AtMYB14, AtMYB15 and AtMYBS3 participated in freezing tolerance in Arabidopsis by repressing the expression of CBF (C‐REPEAT BINDING FACTOR) genes (Agarwal *et al*., 2006a; Chen *et al*., 2013; Su *et al*., 2010). Overexpressing of *TaMYB33* (*Triticum aestivum*) in Arabidopsis led to the down‐regulation of AtABF3 and AtABI1, and the up‐regulation of AtAAO3 (Qin *et al*., 2012). In apple, MdMYB88 and MdMYB124 positively regulated the expressions of cold hardiness and cold‐responsive genes under cold stress by CBF‐dependent and CBF‐independent pathways (Xie *et al*., 2017). In this study, the transcription levels of *MdRD22*,* MdRD29A*,* MdAREB1A* and *MdDREB2A* were positively correlated with that of *MdMYB46* in the wild and transgenic apple under salt and osmotic stress. EMSA, luciferase reporter assays and ChIP further confirmed that MdMYB46 could bind to the SMRE, M46RE and MYBCORE motifs in the promoter of the four genes and activate their expression.

**Figure 7:**
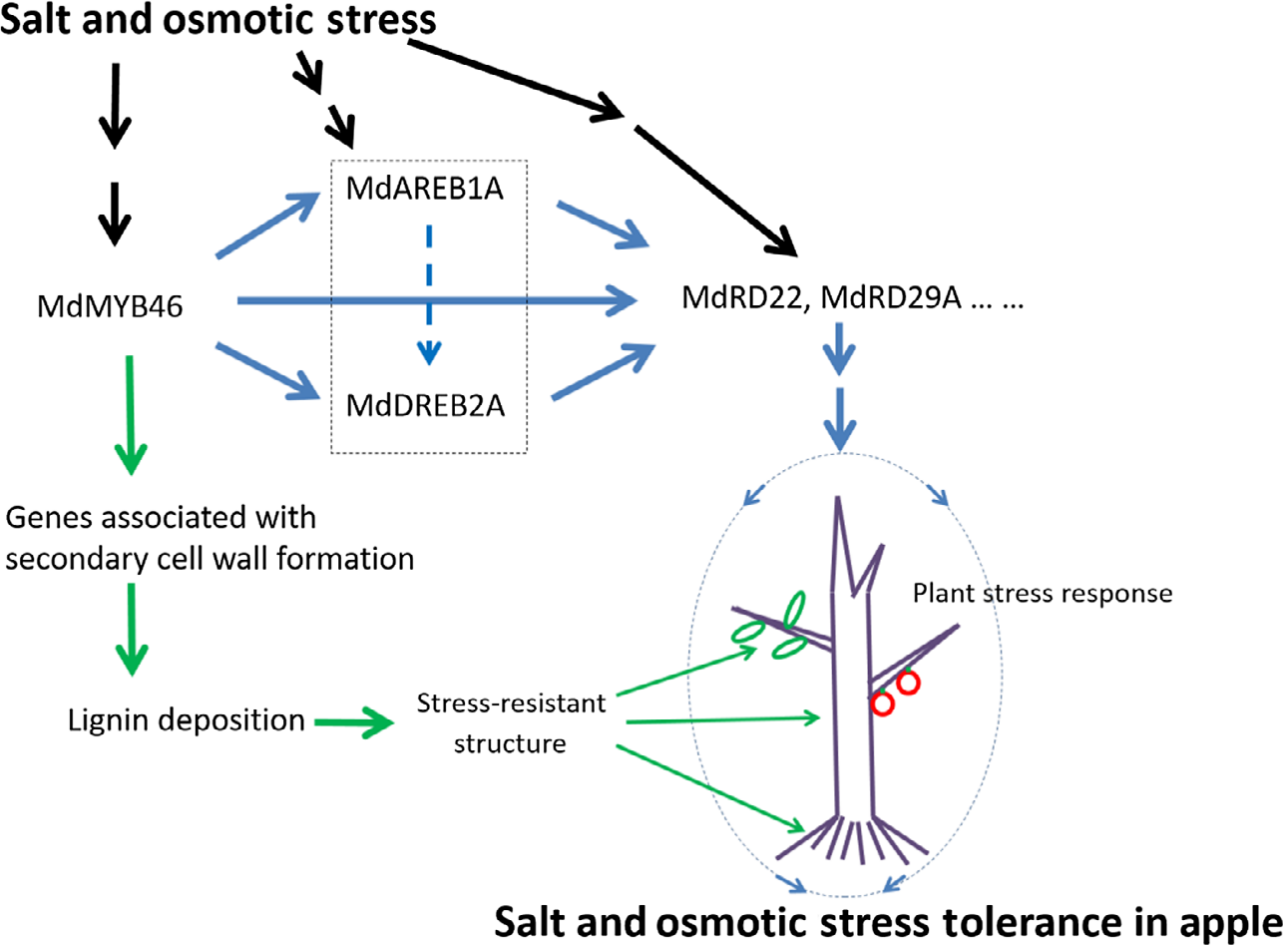
A model for salt and osmotic stress tolerance in apple mediated by MdMYB46. Under stress, MdMYB46 can enhance the salt and osmotic stress tolerance of apples not only by activating secondary cell wall biosynthesis pathways in various organs, but also by activating ABA‐dependent and ABA‐independent stress signals, which may inspire other defence responses in plants. The solid black line represents the upstream stress signal transduction pathway, the green solid line represents the secondary cell wall biosynthesis pathway, and the blue solid line represents the ABA‐dependent and ABA‐independent stress signals pathway. The blue dashed line indicates that there may be an activation reaction, and the dashed box represents an overall synergistic reaction. [Colour figure can be viewed at wileyonlinelibrary.com]

ABA‐dependent and ABA‐independent signalling pathways play signal transduction roles during plant adaptation to osmotic stress (Vishwakarma *et al*., 2017; Yoshida *et al*., 2014; Zhu, 2016), which are closely related to the active oxygen scavenging system and the production of antioxidants in plants (Krasensky and Jonak, 2012; Zhao *et al*., 2018). So, the activation of *MdABRE1A*,* MdDREB2A*,* MdRD22* and *MdRD29A* might be beneficial to stimulate the anti‐stress system in the whole plant, improve the level of stress‐resistant components (such as proline) and enhance the stress tolerance in apple. In addition to regulating the formation of secondary cell wall, MdMYB46 could also enhance the salt and osmotic stress tolerance of apple plants by directly activating *MdABRE1A* in the ABA‐dependent pathway, *MdDREB2A* in the ABA‐independent pathway and dehydration‐responsive genes *MdRD22* and *MdRD29A* (Figure 7).

Jeong *et al*. (2018) found that the expression level of *AtSND1* in *Arabidopsis* was significantly increased under stress treatment conditions, such as mannitol and NaCl. SND1 could coordinate the relationship between plant growth and ABA metabolism by regulating the expression of *ABI4* and *MYB46*. Under the stress simulated by mannitol and NaCl, the increase of *MdMYB46* expression in apple may also be activated by MdSND1. MdSND1, the key upstream transcription factor of MdMYB46 involved in lignin biosynthesis, was identified in apple and its expression was also induced by ABA, salt and mannitol. The stress treatment experiment of transgenic apple lines indicated that MdSND1 could also enhance the salt and osmotic stress tolerance in apple (unpublished date). In further studies, the transcriptional regulation mechanism of MdSND1 on *MdMYB46* and stress response pathways under salt and osmotic stress conditions will be a potential direction to analyse the stress response mechanism in apple. In addition, drought stress could induce the expression of *MdMYB88* and *MdMYB124* in apple, which can activate the transcription of *MdMYB46* in direct or indirect ways (Geng *et al*., 2018). A similar regulation mechanism may happen under our mannitol and NaCl treatment.

In summary, MdMYB46 can enhance the salt and osmotic stress tolerance of apple not only by activating secondary cell wall biosynthesis pathways, but also by directly activating stress‐responsive signals.

## Materials and methods

### Plant materials

The tissue culture plants of apple (*Malus×domestica* Borkh.) used for transformation and resistance treatment were named GL‐3 with high efficiency for transformation (Dai *et al*., 2013), which were cultured in the subculture medium (MS medium supplemented with 0.3 mg/L 6‐BA, 0.2 mg/L IAA, 0.1 mg/L GA_3_) and managed under long‐day conditions (14 h : 10 h, light : dark) at 25 °C. The callus of ‘Ourin’ apple for transformation was kept in our laboratory at 25 °C in the dark, and tobacco used for subcellular localization and transient expression of genes was *Nicotiana benthamiana*. The *Arabidopsis thaliana* was managed under long‐day photoperiod (16 h : 8 h, light : dark) at 23 °C.

### Genetic transformation

The *MdMYB46* full‐length coding sequence (CDS) region [the same to the sequence of MD03G1176000 in the Apple Genome (GDDH13 V1.1) database] amplified with MdMYB46‐pRI primer pair (Table S1) was inserted into the pRI101‐AN vector to construct the overexpression vector of pRI‐MdMYB46 (Figure S2A). Forward and reverse fragments of *MdMYB46* gene were amplified with the RNAi‐MdMYB46 ZF/ZR and FF/FR primer pairs (Table S1), respectively, and inserted into the pRNAi‐E vector (Song *et al*., 2017) to construct the MdMYB46‐RNAi vector (Figure S2A). The overexpression and RNAi vectors of *MdMYB46* were introduced into the *Agrobacterium tumefaciens* strain EHA105. The genetic transformation of GL‐3 was according to the method of Dai *et al*. (2013).

For callus transformation, the MYC tag was added before the *MdMYB46* full‐length CDS and inserted into the pRI101‐AN vector, labelled as pRI‐MYC‐MdMYB46. The fusion vector was introduced into EHA105. The ‘Ourin’ callus, subcultured in MS medium supplemented with 1.5 mg/L 2,4‐D and 0.4 mg/L 6‐BA, was transferred into the bacterial solution, oscillating cultivated for 15 minutes, and transferred to fresh callus medium for pre‐culture. After 2–4 days of dark culture at room temperature, the pre‐cultured callus was transferred to callus screening medium (MS medium supplemented with 1.5 mg/L 2,4‐D and 0.4 mg/L 6‐BA, 250 mg/L cefotaxime and 30 mg/L kanamycin, pH = 5.8). After about 30 days of dark culture, the newly grown callus was transferred to a new screening medium and positive callus was screened for subsequent chromatin immunoprecipitation (ChIP).

For *A. thaliana* transformation, the overexpression vector of *MdMYB46* was introduced into *Agrobacterium* strain GV3101. *A. thaliana* (Col‐0) was transformed using the floral dip method. The seeds collected were screened in 1/2 MS medium supplemented with 30 mg/L kanamycin. Then, the seedlings were transplanted into the matrix for phenotypic observation and those with the similar changes were chosen for further qRT‐PCR detection. The screened T_1_ transgenic lines were used for further analysis.

### Stress treatment

For the short‐time stress treatment, the top young part of the GL‐3 apple plants that has been subcultured for 20 days was cut and then transferred to a liquid subculture containing 50 μm ABA, 200 mm NaCl or 300 mm mannitol. After 0, 2, 4 and 6 h, young tissues were taken for RNA extraction.

For the long‐time stress treatment, the young shoot tip of the 20‐day‐old apple tissue‐cultured plants was cut and transferred to the subculture medium containing 200 mm NaCl or 300 mm mannitol. After 10 days of the treatment, the samples were photographed and their leaves were collected for further tests.

### Measurement of lignin, proline, H_2_O_2_, MDA and relative water content

The stem tissue of 1‐month‐old apple tissue culture plants was used to detect lignin content according to the method of Wang *et al*. (2016). The leaf relative water content was calculated according to the method of Farooqi *et al*. (2000). The proline content was measured spectrophotometrically by the method of Bates *et al*. (1973). The H_2_O_2_ level was measured as described by Velikova *et al*. (2000). Thiobarbituric acid (TBA) test was used to determine the malondialdehyde (MDA) content in leaf with the method described by Heath and Packer (1968).

### NBT and DAB staining

The 3,3′‐diaminobenzidine (DAB) and nitrotetrazolium blue chloride (NBT) staining were performed according to the method described by Kumar *et al*. (2014).

### ChIP Assay

The positive callus transformed with pRI‐MYC‐MdMYB46 of ‘Ourin’ was screened. After the positive callus was grown for 20 days, the ChIP experiment was performed. The main steps were based on the instructions of EPIGENTEC's Plant ChIP kit (Farmingdale). The formaldehyde was firstly used to cross‐link with the chromatin in the ‘Ourin’ callus overexpressing *MYC‐MdMYB46*, and then, MYC immuno‐antibody was used to precipitate and enrich the DNA fragment bound to MYC‐MdMYB46. The immunoprecipitated DNA fragments were then used as templates in qRT‐PCR for detection of *MdMYB58*,* MdMYB63*,* MdCAD*,* MdCOMT*,* MdCCR*,* MdAREB1A*,* MdDREB2A*,* MdRD22* and *MdRD29A* promoter sequences by ABI 7500 real‐time PCR instrument. We used the *MdACTIN* as the internal reference gene, and the 2^−△△Ct^ method (Livak and Schmittgen, 2001) was used to calculate the fold enrichment of each promoter fragment with ABI 7500 software v2.0.6. All primers are listed in Table S1.

### Promoter binding site analysis

Promoter sequences (2000 bp) of *MdMYB58*,* MdMYB63*,* MdC4H*,* MdC3H*,* MdCAD*,* MdF5H*,* MdAREB1A*,* MdDREB2A*,* MdRD22* and *MdRD29A* were downloaded from the Apple Genome website (https://iris.angers.inra.fr/gddh13/jbrowse/?data=gddh13) and analysed whether there were SMRE (ACC[A/T]A[A/C][T/C]), M46RE ([A/G][G/T]T[A/T]GGT[A/G]), MYBCORE (CAGTTA, CTGTTG), MYBR (TGGTTAG), ABRE (ACGTGGC, ACGTGTC) and DRE/TRE (G/ACCGAC, GGCCGACAT) elements. The sequence of cis‐elements is listed in Table S2. The number of each gene is listed in Table S3.

### EMSA

The full‐length CDS region of *MdMYB46* was inserted into the prokaryotic expression vector pGEX‐5X‐1 and labelled as pGEX‐MdMYB46. Then, the constructed vector was separately introduced into the competent cells of *Escherichia coli* BL21 to induce protein production. The GST protein was purified according to the instructions of the Thermo GST Fusion Protein Purification Kit (Massachusetts). The EMSA probe was prepared according to the Biyuntian Biotin Probe Labeling Kit (Shanghai, China), and the fusion between protein and labelled probes was made with reference to the EMSA kit (Thermo, Rockford, IL, USA). The non‐denaturing polyacrylamide gel was prepared, and the test was completed by spotting, electrophoresis detection, film transfer, film washing and development.

### Analysis of transcriptional activity

The constructed pRI‐MdMYB46 vector was used as an effector. The clipped region (about 200‐500 bp fragment containing the corresponding binding site) of *MdMYB58*,* MdMYB63*,* MdC4H*,* MdC3H*,* MdCAD*,* MdF5H*,* MdAREB1A*,* MdDREB2A*,* MdRD22* or *MdRD29A* was inserted into the vector of pRI‐mini35S‐LUC and used as a reporter. The conserved bases of the binding sites in the *MdAREB1A*,* MdDREB2A*,* MdRD22* and *MdRD29A* promoter fragments were mutated by PCR and ligated into the vector of pRI‐mini35S‐LUC as a control.

The above‐constructed vector was transferred into the leaves of 1‐month‐old *N. benthamiana* for transient expression. The infected tobacco was cultured for 24 h in the dark and then transferred to a light incubator (25 °C, 16 h : 8 h, light : dark). After 48 h, the tobacco was photographed by a living fluorescence imager (Lb985, Berthold, Germany). The luciferase activity of these leaves was measured using a Transgen Biotech firefly luciferase assay kit (Beijing, China).

### RNA extraction and qRT‐PCR

Total RNA was extracted from leaves using the modified CTAB method (Chang *et al*., 2007), and cDNA was obtained with reference to the Takara Bio PrimeScript^™^ RT Reagent Kit (Dalian, China). The expression level of these genes was detected by ABI 7500 real‐time PCR instrument. The reaction system was in a total volume of 10 μL, containing 0.5 μL of cDNA, 5.0 μL of TaKaRa SYBR Premix Ex Taq, 0.5 μL of upstream and downstream primers for the relevant detection gene (with each primer at 0.2 μm), 0.2 μL 50 × ROX Reference Dye II, and 3.3 μL ddH2O. The amplification programme was as follows: one cycle of 10 min at 95 °C followed by 40 cycles of 10 s at 95 °C and 30 s at 60 °C; and the dissolution curve was plotted at 95 °C for 15 s, 60 °C for 1 min, 95 °C for 30 s and 60 °C for 15 s. All reactions were run in triplicate, and average values were calculated. *MdActin* and *At18S* were used as the internal reference for apple and *Arabidopsis*, respectively, and the 2^−△△Ct^ method (Livak and Schmittgen, 2001) was used to calculate the relative expression level of each gene with ABI 7500 software v2.0.6.

### Subcellular localization

The *MdMYB46* full‐length CDS without the stop codon was amplified with MdMYB46‐eGFP primer pair (Table S1) and inserted into the pRI‐eGFP vector to constitute MdMYB46‐eGFP fusion expression vector. The fusion vector was introduced into EHA105 and transiently expressed in leaves of *N. benthamiana* (1‐month‐old) by infiltration method (Yang *et al*., 2000). The infected tobacco was cultured for 24 h in the dark and then transferred to a light incubator (25 °C, 16 h : 8 h, light : dark). After 48 h, the tobacco leaves were photographed by a confocal laser‐scanning microscope (TCS SP8‐SE; Leica, Wetzlar, Germany).

### Analysis of transcriptional activity of MdMYB46

The full‐length CDS of *MdMYB46* and their N‐terminal DNA‐binding region and C‐terminus were inserted into pGBT9 vector containing the DNA‐binding region of *GAL4*, and were labelled as pGBT9‐MdMYB46, pGBT9‐MdMYB46‐N and pGBT9‐MdMYB46‐C, respectively. The vector plasmid was transferred to the Y2H strain and cultured according to the manufacturer's instructions (Clontech). The yeast on the selective medium lacking Trp (‐T) solid medium plate was activated onto the medium lacking Trp, His and adenine (‐T/‐H/‐A) + X‐alpha‐gal (5‐Bromo‐4‐chloro‐3‐indolyl‐α‐D‐galactoside) solid medium plate. If the yeast grew and turned blue, it was indicated that the gene had transcriptional activation activity.

## Conflict of interest

The authors have no conflict of interest to declare.

## Author contributions

Hongyan Dai and Zhihong Zhang conceived the research plan and supervised the experiments; Keqin Chen, Mengru Song and Yunna Guo performed the most of the experiments and analyse the data; Lifu Liu and Hao Xue assisted the management of the transgenic plants; and Keqin Chen wrote the article.

## Supporting information


**Figure S1** Multiple sequence alignments and phylogenetic analysis of MdMYB46 orthologs. **Figure S2** Transcriptional levels of *MdMYB46* in transgenic apple. **Figure S3** Phenotypes of *MdMYB46*‐overexpressing Arabidopsis and apple. **Figure S4** Differences in transcriptional levels of lignin biosynthesis‐related genes between *MdMYB46*‐overexpressing Arabidopsis plants and wild type. **Figure S5** Changes in expression of genes related to cellulose biosynthesis in *MdMYB46*‐overexpressing Arabidopsis and apple. **Figure S6** Transcriptional levels of stress signaling genes under ABA, salt and drought stress and in *MdMYB46* transgenic apples. **Table S1** Primers used in this study. **Table S2** Sequence of cis‐elements in this study. **Table S3** Accession number of each gene in this study. **Table S4** Partial study on genes related to stress response in apple.
