# Supplementary material for: MdMYB46 could enhance salt and osmotic stress tolerance in apple by directly activating stress‐responsive signals
Source: Plant Biotechnol J. 2019 May 26;17(12):2341–55. doi: 10.1111/pbi.13151 (PMC6835124; doi:10.1111/pbi.13151)
Supplement: Supplementary file 1 — Figure S1 Multiple sequence alignments and phylogenetic analysis of MdMYB46 orthologs. Figure S2 Transcriptional levels of MdMYB46 in transgenic apple. Figure S3 Phenotypes of MdMYB46‐overexpressing Arabidopsis and apple. Figure S4 Differences in transcriptional levels of lignin biosynthesis‐related genes between MdMYB46‐overexpressing Arabidopsis plants and wild type. Figure S5 Changes in expression of genes related to cellulose biosynthesis in MdMYB46‐overexpressing Arabidopsis and apple. Figure S6 Transcriptional levels of stress signaling genes under ABA, salt and drought stress and in MdMYB46 transgenic apples. Table S1 Primers used in this study. Table S2 Sequence of cis‐elements in this study. Table S3 Accession number of each gene in this study. Table S4 Partial study on genes related to stress response in apple. [file PBI-17-2341-s001.pdf]

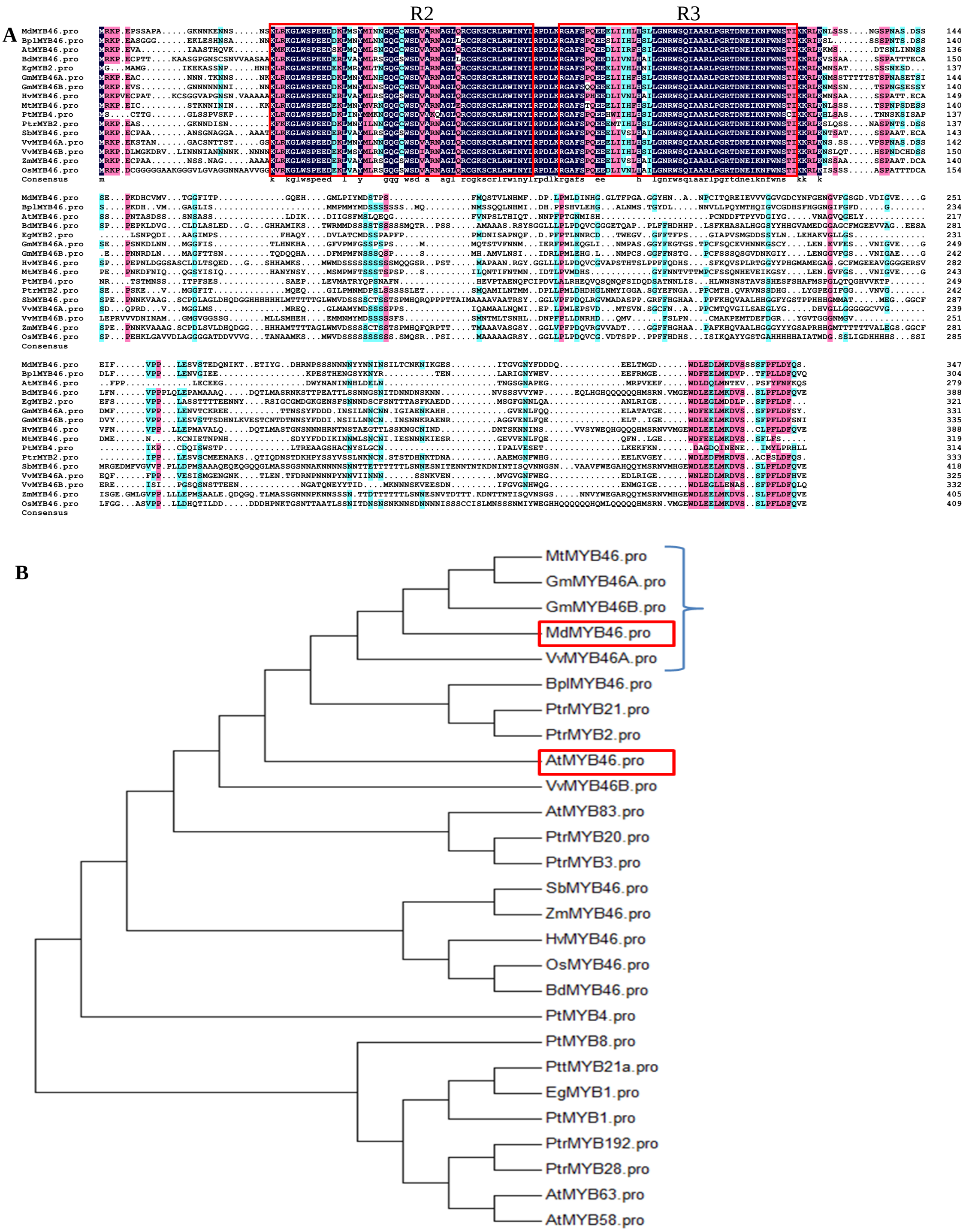

Supplemental Figure S1. Multiple sequence alignments and phylogenetic analysis of MdMYB46 orthologs. A. Multiple sequence alignments of AtMYB46 orthologs in *Eucalyptus* (*Eucalyptus grandis*; EgMYB2); Pine (*Pinus taeda*; PtMYB4), grapevine (*Vitis vinifera*; VvMYB46), alfalfa (*Medicago truncatula*; MtMYB46), soybean (*Glycine max*; GmMYB46), rice (*Oryza sativa*; OsMYB46), maize (*Zea mays*; ZmMYB46), sorghum (*Sorghum bicolor*; SbMYB46), barley (*Hordeum vulgare*; HvMYB46), brachypodium (*Brachypodium distachyon*; BdMYB46), birch (*Betula platyphylla*, BplMYB46) and apple (*Malus*×*domestica* Borkh, MdMYB46). R2, R3 functional regions were marked in the red box. B. Phylogenetic analysis of AtMYB46/83 orthologs. MYB58, MYB63 and their homologs (PtMYB1/8/21a, PtrMYB28/192 and EgMYB1) are included as the outgroup. AtMYB46 and MdMYB46 were marked with red frame. Proteins in the blue half brackets represent the members in the same subfamily with MdMYB46.

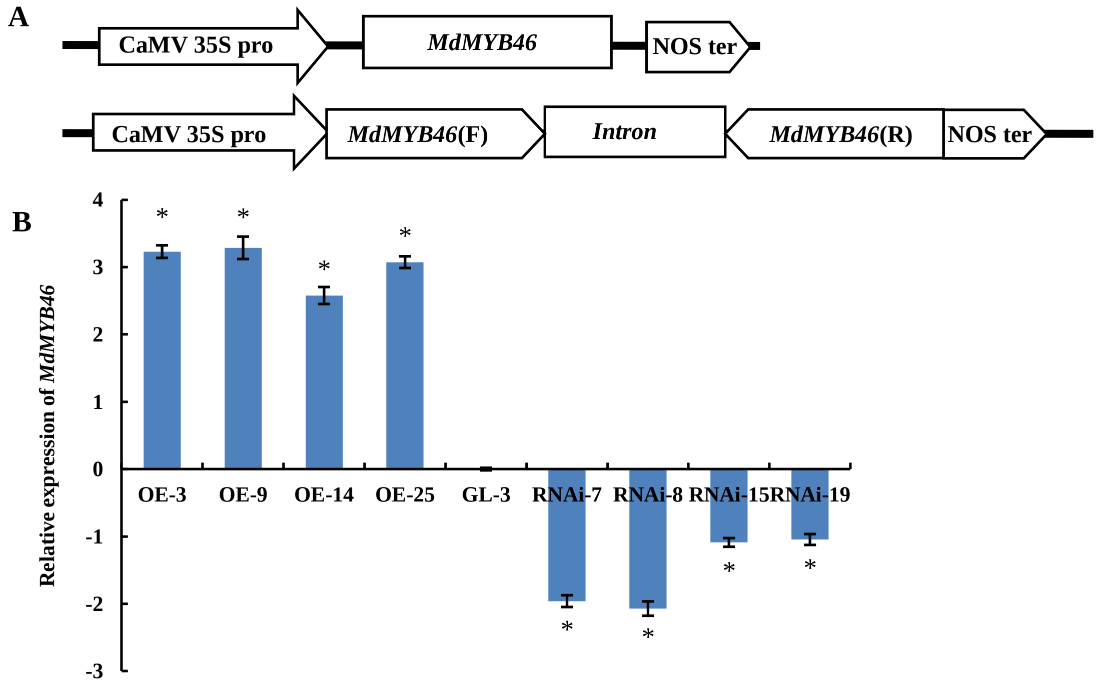

Supplemental Figure S2. Transcriptional levels of *MdMYB46* in transgenic apple. A. Schematic shown the overexpressing and RNAi vector of *MdMYB46*. B. *MdMYB46* expression levels in non-transgenic GL-3 apple plants, *MdMYB46*-overexpressing apple plants (OE-MdMYB46) and *MdMYB46*-RNAi plants (RNAi-MdMYB46). The error bars indicate the standard deviation (SD) from three biological replicates. Asterisk indicates significant differences between the transgenic lines and GL-3 ($P < 0.05$, based on t-test).

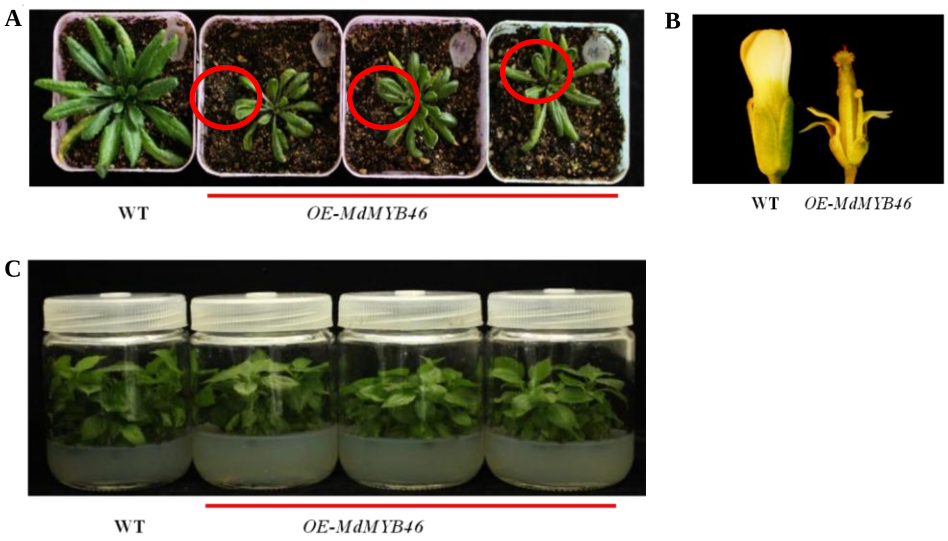

Supplemental Figure S3. Phenotypes of *MdMYB46*-overexpressing Arabidopsis and apple. A. Phenotypic differences between *MdMYB46*-overexpressing Arabidopsis plants and non-transgenic wild type (WT). The curling blade was marked in the red box. B. Differences in petal structure between *MdMYB46*-overexpressing Arabidopsis and WT. C. Phenotype of *MdMYB46*-overexpressing apple plants and WT.

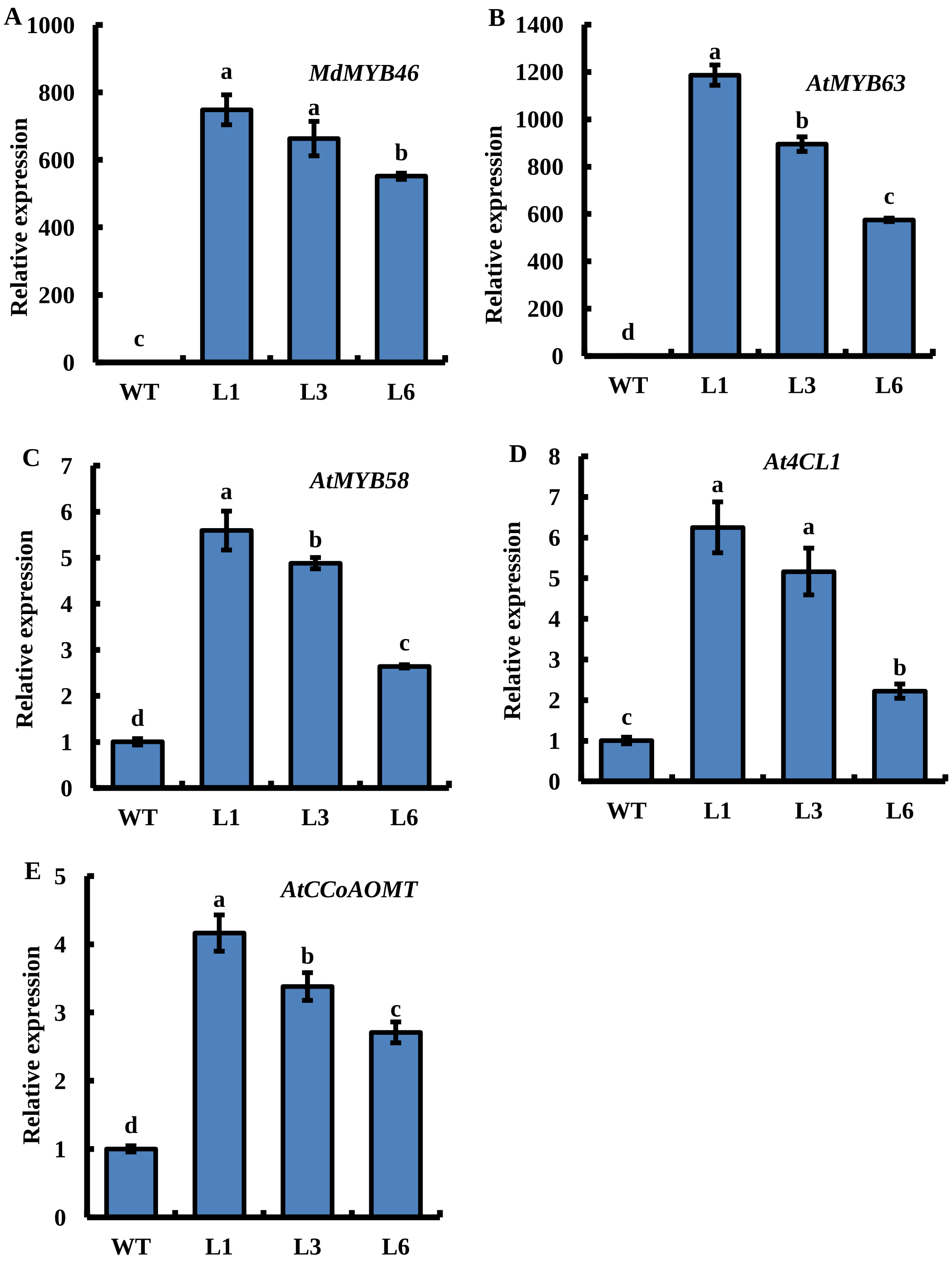

Supplemental Figure S4. Differences in transcriptional levels of lignin biosynthesis-related genes between *MdMYB46*-overexpressing Arabidopsis plants and wild type. A-E. The error bars indicate the standard deviation (SD) from three biological replicates. Different letters indicate significant differences ($P < 0.05$, based on Duncan's multiple range test). WT represents the non-transgenic wild type while L1, L3 and L6 represent the transgenic lines.

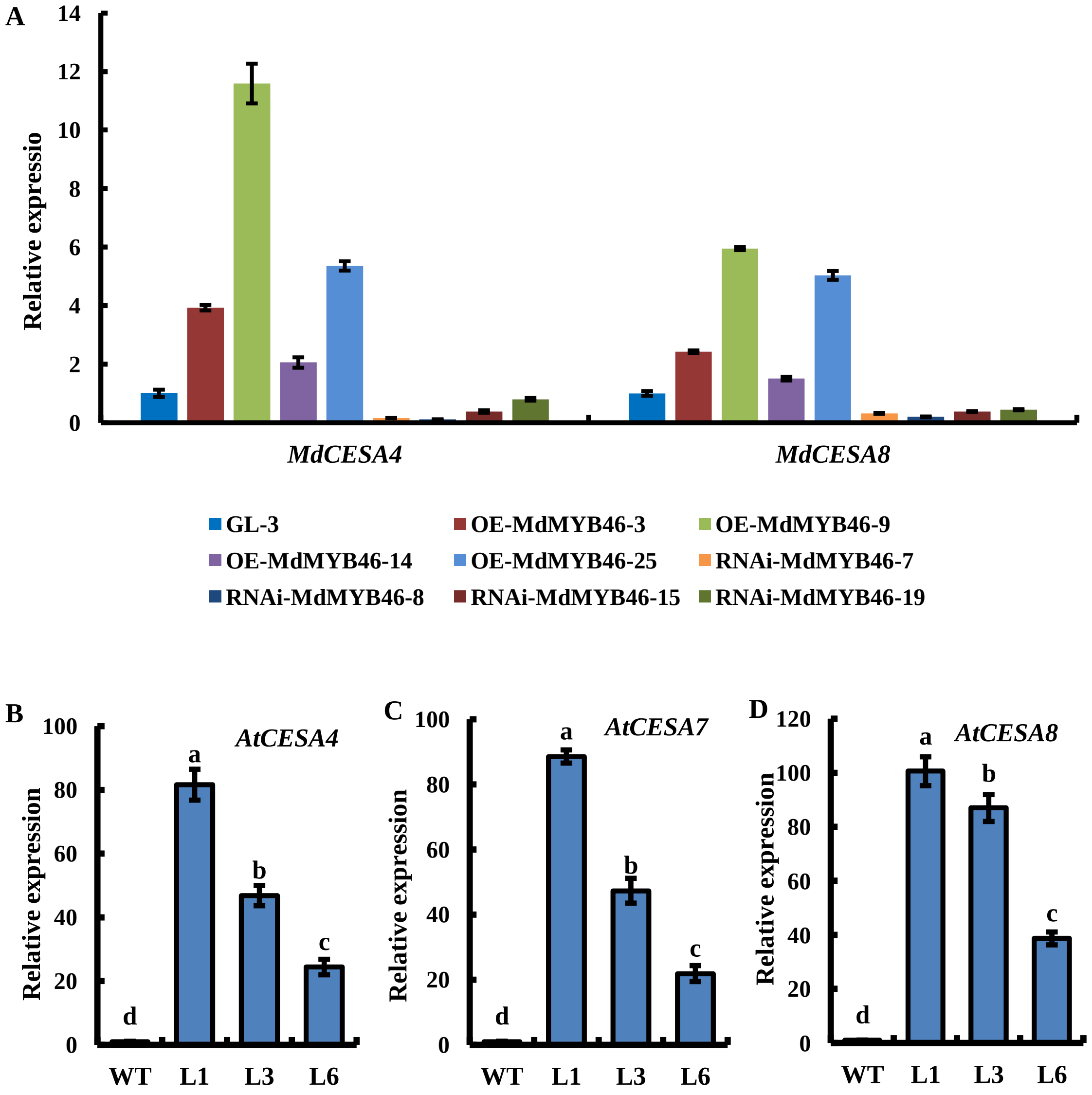

Supplemental Figure S5. Changes in expression of genes related to cellulose biosynthesis in *MdMYB46*-overexpressing Arabidopsis and apple. A. Transcriptional levels of cellulose biosynthesis-related genes in *MdMYB46*-overexpressing apple plants and non-transgenic plants (GL-3). B-D. Transcriptional levels of cellulose biosynthesis-related genes in *MdMYB46*-overexpressing Arabidopsis plants and the non-transgenic wild type (WT). The error bars indicate the standard deviation (SD) from three biological replicates. Different letters indicate significant differences ($P < 0.05$, based on Duncan's multiple range test).

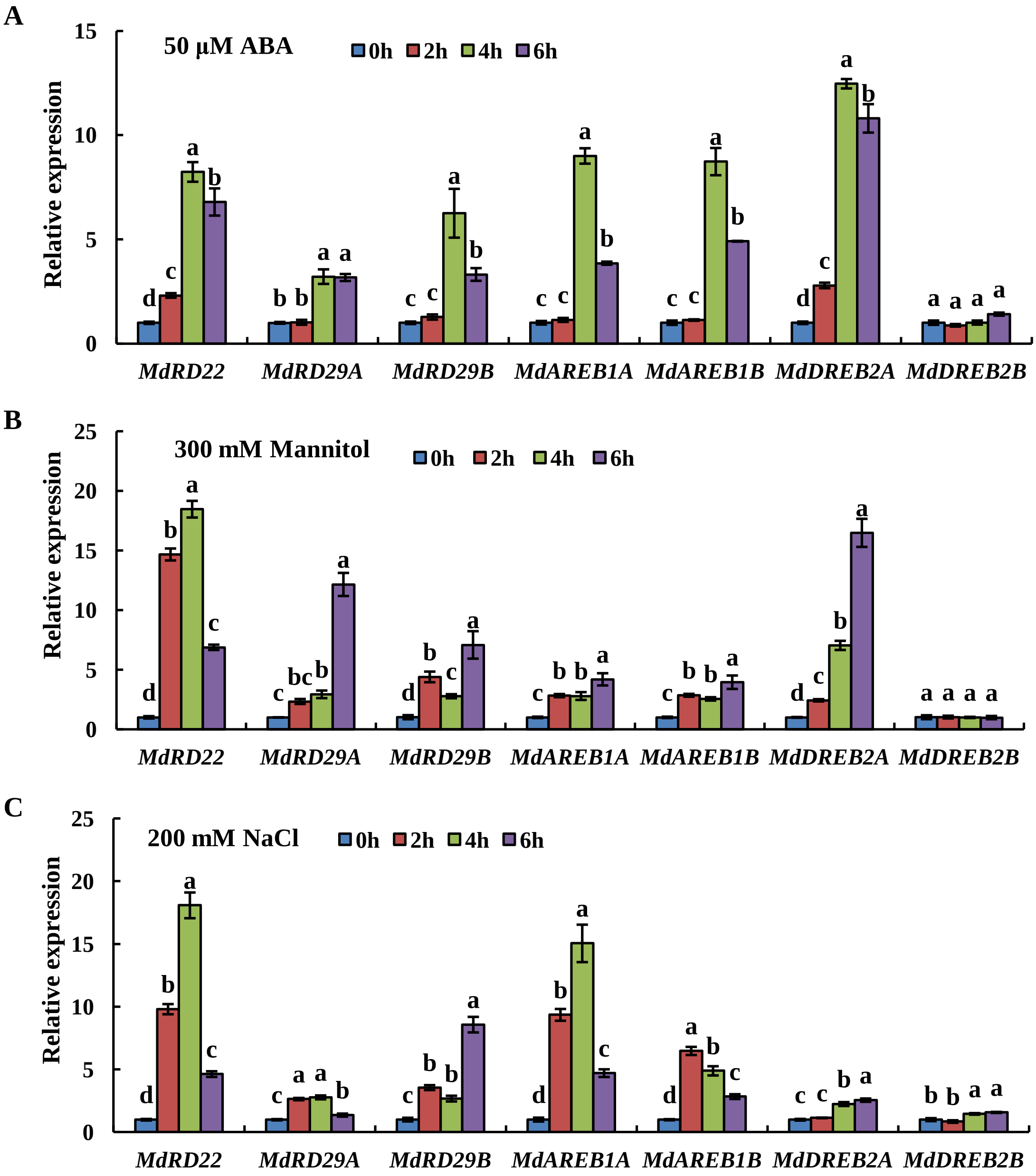

Supplemental Figure S6. Transcriptional levels of stress signaling genes under ABA, salt and drought stress and in *MdMYB46* transgenic apples. A. B. C. The expression levels of stress signaling genes in non-transgenic GL-3 apple plants under ABA, NaCl and mannitol treatment. The error bars indicate the standard deviation (SD) from three biological replicates. Different letters indicate significant differences (*P* < 0.05, based on Duncan's multiple range test).

Supplemental Table S1 Primers used in this study

Primer Name	Primer Sequence
**For transgenic vectors**	
MdMYB46-eGFP	F: GTCGACATGAGGAAGCCAGAACCCTCC
	R: GGATCCACTTTGGTAGTCAAGAAAAGG
MdMYB46 (Y2H)	F:GGATCCTGAGGAAGCCAGAACCCTCC
	R:GTCGACTCAACTTTGGTAGTCAAGAAA
MdMYB46-N	F:GGATCCTGAGGAAGCCAGAACCCTCC
(Y2H)	R:GTCGACATGATCCTTAGGCTCTGAGGA
MdMYB46-C	F:GGATCCGTGTGATGGTGACCGGAGGG
(Y2H)	R:GTCGACTCAACTTTGGTAGTCAAGAAA
MdMYB46- pRI	F: GTCGACATGAGGAAGCCAGAACCCTCC
	R: GGATCCATGAGGAAGCCAGAACCCTCC
MdMYB46-	F: GGATCCCCATGAGGAAGCCAGAACCCTCC
pGEX	R: GTCGACACTTTGGTAGTCAAGAAAAGG
MYC-MdMYB46-	F: GTCGACATGGAACAAAAGTTGATTTCTGAA
pRI	GAAGATCTTATGAGGAAGCCAGAACCCTCC
	R: GGATCCATGAGGAAGCCAGAACCCTCC
RNAi-MdMYB46	ZF:CTCGAGGATGGTGACCGGAGGGTT
	ZR:GAATTCTCCCCATAGATGGTTTCA
	FF:TCTAGATCCCCATAGATGGTTTCA
	FR:AAGCTTAACCCTCCGGTCACCATC
**For EMSA probes**	
pMdMYB58	F: TGGTGCTTTGAGATTATTAGGTGATTTGGAGCCGCAAT
	R: ATTGCGGCTCCAAATCACCTAATAATCTCAAAGCACCA
pMdMYB63	F: TTTGTTGTGAGGTCAATTTGGTAGTTTCCACACCAAAG
	R: CTTTGGTGTGGAAACTACCAAATTGACCTCACAACAAA
pMdCAD	F: TAATATTAGGAAGACCAAATTTTTAAACTAAAT
	R: ATTTAGTTTAAAAATTTGGTCTTCCTAATATTA
pMdCOMT	F: ACACAATAGTCAACCTAATTTGGTATTGAGTTTGTCA
	R: TGACAAACTCAATACCAAATTAGGTTGACTATTGTGT
pMdCCR	F: AACCCCACTAATAACACCAACTAAAATATCATTTTTT
	R: AAAAAATGATATTTTAGTTGGTGTTATTAGTGGGGTT
pMdRD22	F: ATCTTAATTAGGTGTGTCGAGACATCTTGCAATAAAA
	AACCAACTCATATT

	R: AATATGAGTTGGTTTTTATTGCAAGATGTCTCGACACA CCTAATTAAGAT
pMdRD29A	F: AAAATTTGATTTACCTAACATTACTACTTAT
	R: ATAAGTAGTAATGTTAGGTAAATCAAATTTT
pMdAREB1A	F: AATTCAACCTAACTTTAATCCGTTTATTTATTGGATTGAG TTGGGTTGGTGATTTGG
	R: CCAAATCACCAACCCAACTCAATCCAATAAATAAACGGATT AAAGTTAGGTTGAATT
pMdDREB2A	F: AAAGCGCAAAAAACCAAACCAGCAGAAAAAG
	R: CTTTTTCTGCTGGTTTGGTTTTTTGCGCTTT
**For reporter genes**	
pMdMYB58	F: CTGCAGATGGTGTTGGCCAATTTTACA
	R: GGATCCCAGTAATCATAAGGTTGCAAG
pMdMYB63	F: CTGCAGTTACTCTATAGAGGTGGTGCT
	R: GGATCCTAGTGAGATGGGAAAAACGTC
pMdCAD	F: CTGCAGCGAATAAATTGAAGAAGATTA
	R: GGATCCGACACATTATTTAATATTAGA
pMdCOMT	F: CTGCAGGGTGGTGCTGCATGATCATG
	R: GGATCCTGGGCACACAAGGCAAGTAAT
pMdCCR	F: CTGCAGAACCCCTATGATTGCATTCAC
	R: GGATCCTCGAGAGAAGAGACAGACAGA
pMdRD22-P4	F: CTGCAGATTGATAAACCAATTCAAGAT
	R: GGATCCCCTGTTTTGTGTGGTCACGTA
pMdRD29A-P1	F: CTGCAGTGCGAGAGCCCTAGGATCTCC
	R: GGATCCCTAGGGTTACAACATTTGACA
pMdAREB1A-P2	F: CTGCAGGAATAGTATCAGGATTGACTT
	R: GGATCCATCATTTATTGTTCAAATGAA
pMdDREB2A-P1	F: CTGCAGCACAATCGCAAATTTCCGAAT
	R: GGATCCCAAACTCAACCAACCTCAGAA
pMdRD22m	F: TAATTAAGTGTGTCGAGACATCTTGCAATAA AAAGCCAACTCA
	R: TGAGTTGGCTTTTTATTGCAAGATGT CTCGACACACTTAATTA
pMdRD29Am	F: CTAAAATTTGATTTGCCTAACATTACTACTTATAT
	R: ATATAAGTAGTAATGTTAGGCAAATCAAATTTTAG

pMdAREB1Am	F: CAGCCTAACTTTAATCCGTTTATTTATTGGATTGAGT TGGGTTAGTGAT
	R: ATCACTAACCCAACTCAATCCAATAAATAAACGGATTAAA GTTAGGCTG
pMdDREB2Am	F: GCAAAGCGCAAAAAGCCAAACCAGCAGAAAAAGGC
	R: GCCTTTTTCTGCTGGTTTGGCTTTTTGCGCTTTGC
**For ChIP-PCR**	
pMdMYB58	F: AGTCTCGCTATATGGTATATG
	R: GGTACTCTAAAACAATTATAG
pMdMYB63	F: AAGAGTTGCTTGTGTTTTGCA
	R: TCATGATTTTAAAGCATCGTT
pMdCAD	F: CCCAATTCGGGTATGCAAAAT
	R: GGCAAACCGAATGATGTGTCA
pMdCOMT	F: AGTACATTTTTACTAAGAGGG
	R: ACGATGTGGAGTCTATAGTAT
pMdCCR	F: AGTCATTTACAACCCCTATGA
	R: ATGTGACAGGAGTAACATGAC
pMdRD22-P1	F: ATGTGTCCTTTAACTTAACCA
	R: TGTAGGAGCAAAGGGAGACG
pMdRD22-P2	F: GTTAACAGAGTTATATCTATG
	R: GGACTGGTCATTGGTTTGTAC
pMdRD22-P3	F: CAATGACCAGTCCAAGCTATA
	R: GATGTTGATTTATGGCATTCA
pMdRD22-P4	F: ACTAAATTCAAGCACCCATGT
	R: CACCGGAAATGAAGGGCAGAC
pMdRD29A-P1	F: GATGTAGTTGTTGATAATTAA
	R: CTAGGGTTACAACATTTGACA
pMdRD29A-P2	F: TCCTCTATCCTTACCTATCTC
	R: TCTGTCGCTCTGGGAGCGAGA
pMdAREB1A-P1	F: GCTGCGAAGACCCGTTTGATT
	R: AGTCAATCCTGATACTATTCA
pMdAREB1A-P2	F: GAATAGGTCTATGTCTATTTT
	R: TGGACAAATAACATGCATGAG
pMdAREB1A-P3	F: CGTTGTCGGCTCACAGCCTTC
	R: TCCACGTTTTCTTGAATGGAG

pMdDREB2A-P1	F: TATCGGAAATATCTAGAGGAA
	R: ATTCGAGAAAGTCGAAGACTG
**For qRT-PCR**	
MdMYB46(DL)	F:CATCCTCACCTGCAATAAAAA
	R:GGTAGTCAAGAAAAGGAAATG
MdMYB58(DL)	F: TGGCTTAGTTACTTGGAAAGT
	R: TGAATGGAGGAATTGTTGTGT
MdMYB63(DL)	F: GTTACTTGCAAAGTGAGCTTG
	R: AAGAAAGCCACGTTTGAAAGT
MdC4H(DL)	F: ACATGAACCTCCAGGATGCCA
	R: GAGATACCTGAAGTCGTTCCC
MdC3H(DL)	F: CTACTTCCATTTGGAGCAGGC
	R: CGGAGTAGGCACGACTTGGAC
MdCAD(DL)	F: ATGCAAGAAGCTGCTGACTCA
	R: GAAGCTCCCTGTGATTGTCTT
MdF5H(DL)	F: AGCCCTCTAGGTTTCTGAAAG
	R: GTCAAGCTCACTAGGTTTCAT
MdHCT(DL)	F: TGCTTTGGTGCGTATGGATGA
	R: CCAAAGTCGGCATCATGGATT
Md4CL(DL)	F: CCCTGATACGGGTGCTTCGCT
	R: CGGTCGACGATGAAGAGCTCG
MdCOMT(DL)	F: GGTGAAGGTTGGGGGTTTGAT
	R: GATTCGACGGCAGAGAGTGAT
MdCCR	F: TCACATTCTGGTCTATGAGAC
	R: CTTCTGGTTTGTGAACTTGTA
MdCESA4	F:TGGTACTTTGGTCAGTTCTTC
	R:ATCCCAATATACACCCGAGTT
MdCESA8	F:GGCCTCTGTCTTCTCCCTTGT
	R:CTCACATGTGATTCCCAGAGA
MdRD22(DL)	F：TGATGGGGTGAATGTTAAAG
	R：GAACCCAGACAACATGATCA
MdRD29A(DL)	F：CTGAAGAAGGTAAAGGAGAA
	R：CCTTCAAAATATCTCCTTGC
MdRD29B(DL)	F：CCAAATTACCATGCCTCAAC
	R：CCTTGGACTTGTACTCTCCC

MdAREB1A(DL)	F：CAGAGAATCAGCTGCCAGGT
	R：TCTCCATGTCCTGATTCTTC
MdAREB1B(DL)	F：TTAGAACTAGAGGCAGAAGT
	R：CTGTCAATGTTCGTCGTAAG
MdDREB2A(DL)	F：AAGAAAAGGAGGGGAGTAAT
	R：ATAGTTGTAACCTCCATCTC
MdDREB2B(DL)	F：CTCTAATTCTGTGTCATCTA
	R：AACATGTAATTCCTCTGATA
MdActin	F: TGGTGAAGGCTGGATTTG
	R: CTGTGAGCAGAACTGGGTG
At4CL	F:ATGATGTGAAGCAATTCGTGT
	R:GGTTGGGTCAAACAGTTGTAT
AtCCOAOMT	F:GAAAATTGGAGGAGTGATTGG
	R:CCACTGTGGACAAACTCAGAG
AtCESA4	F: GGGTCATTGTTCATCTTT
	R: TGCGGTTCCTCATTTAAC
AtCESA7	F: TAGACAGAACAGAACACC
	R: CACTCTCGACAAAGTACA
AtCESA8	F: CCTTTTGGGTGATTCTTC
	R: CGTATCTTCTCTTAGCAA
AtMYB46	F:ACCACTTAGACGAGTTGAACA
	R:TGTGTGGCTAAGAATCTTGAG
AtMYB58	F:GTTTCATAGATGGTTCAGACTCA
	R:GGTGAAAATGACTTGCTTTATGG
AtMYB63	F:TCAAGAGCAACAACAGAAGAA
	R:TCTCTCTACCTTTCCATCATA
At18S	F:ACACGGGGAGGTAGTGACAA
	R:CCTCCAATGGATCCTCGTTA

Supplemental Table S2 Sequence of cis-elements in this study

Cis-element	Sequence
M46RE	(A/G)(G/T)T(A/T)GGT(A/G)
SMRE	ACC(A/T)A(A/C)(T/C)
SNBE	(T/A)NN(C/T)(T/C/G)TNNNNNNNA(A/C)GN(A/C/T)(A/T）
ABRE	ACGTGGC, ACGTGTC
DRE/TRE	G/ACCGAC, GGCCGACAT
MYBCORE	CAGTTA, CTGTTG
MYBR	TGGTTAG

Supplemental Table S3 Accession number of each gene in this study

**Gene**	**Accession number**
MdMYB46	MD03G1176000
MdMYB83A	MD07G1010500
MdMYB83B	MD02G1312700
MdSND1	MD06G1121400
MdMYB58	MD05G1224100
MdMYB63	MD10G1205400
MdCAD	MD05G1089800
MdCCR	MD17G1222400
MdC3H	MD08G1242900
MdC4H	MD11G1052900
MdCOMT	MD05G1083900
MdF5H	MD02G1136000
Md4CL	MD13G1257800
MdHCT	MD17G1225100
MdAREB1A:	MD15G1081800
MdAREB1B	MD05G1082000
MdDREB2A	MD01G1158600
MdDREB2B	MD04G1165400
MdRD22	MD15G1098800
MdRD29A	MD01G1201000
MdRD29B	MD07G1268800
MdCESA4	MD00G1061100
MdCESA8	MD10G1276500
At4CL	AT1G51680
AtCCoAOMT	AT4G34050
AtCESA4	AT5G44030
AtCESA7	AT5G17420
AtCESA8	AT4G18780
AtMYB58	AT1G16490
AtMYB63	AT1G79180

Supplemental Table S4 Partial study on genes related to stress response in apple

Gene	Accession number	References	Gene name in references
MdAREB1A:	MD15G1081800	Ma et al.,2017	*AREB3.2*
MdAREB1B	MD05G1082000	Ma et al.,2017; Shao et al.,2019	*AREB2*
MdDREB2A	MD01G1158600	Zhao et al.,2012	MDP0000147009
MdDREB2B	MD04G1165400	Zhao et al.,2012	MDP0000153866
MdRD22	MD15G1098800	Shao et al.,2019	*MdRD22*
MdRD29A	MD01G1201000	An et al.,2018	*MdRD29A*
MdRD29B	MD07G1268800	Shao et al.,2019	*MdRD29B*
